# Novel route to enhance the thermo-optical performance of bicyclic diene photoswitches for solar thermal batteries

**DOI:** 10.3762/bjoc.20.93

**Published:** 2024-05-13

**Authors:** Akanksha Ashok Sangolkar, Rama Krishna Kadiyam, Ravinder Pawar

**Affiliations:** 1 Laboratory of Advanced Computation and Theory for Materials and Chemistry, Department of Chemistry, National Institute of Technology Warangal (NITW), Warangal, Telangana-506004, India

**Keywords:** bridged bicyclic diene (BBD), molecular solar thermal (MOST) energy storage, reversible photoswitches, unsaturated bridge elongation

## Abstract

Harnessing solar energy by employing chemical photoswitches in molecular solar thermal (MOST) energy storage systems is a topic of appealing research interest. However, incorporating all the features desired for an ideal MOST system in a single photoswitching couple is challenging. Inspired by experimental synthesis, herein we report our attempt to enhance both the thermochemical and photophysical properties in a single-bridged bicyclic diene (BBD)-based photoswitch by elongating the unsaturated bridge with different heteroatomic units. To elucidate the best elongation unit, the energy storage capacity and the TBR barriers were accounted using the DLPNO-CCSD(T) and (8,8)-CASPT2 methods, respectively. The photophysical properties including the absorption onset, excitation wavelengths, and the absorption intensities were extensively investigated with the time-dependent calculations. The result provides information on the most versatile solvent to exhibit the best photoswitching behaviour which is beneficial for real-life energy storage applications. Additionally, the stability and reversibility of the photoswitching system with elongated unsaturated bridges have also been assessed. By means of the studied modification, the storage energy of 158.57 kJ/mol, energy storage density of 1.48 MJ/kg, TBR barrier of 136.36 kJ/mol, and the absorption onset of 305.00 nm is achieved in acetonitrile. These values are substantially higher when compared with the storage energy (96.06 kJ/mol), energy storage density (1.04 MJ/kg), and TBR barrier (121.76 kJ/mol) of prototype NBD/QC in the gas phase. The outcomes render useful insights into the stability and properties of bicyclic diene-based photoswitches having elongated unsaturated bridges and indeed paves the way for the rational design of practical MOST systems.

## Introduction

Energy is an inevitable necessity of society and its rate of consumption is continuously increasing across the world due to increasing population and technological developments [1–2]. Fossil fuels like natural gas, oil, and coal are currently the chief energy resources whose excessive utilization causes serious environmental issues [2–3]. Further, their reserves are in a threat of being exhausted [3–4]. The sun is the most fundamental and green energy resource that has potential to meet the increasing energy crises in a sustainable manner [2–4]. However, technological devices must be developed that enable a direct capture, conversion, and storage of the solar energy [2,5–7]. Molecular solar thermal (MOST) energy storage is one of the most promising approaches for this purpose [8–13].

The MOST system employs a photoswitchable molecular couple for harvesting, conversion, and storage of solar energy [8–13]. Upon exposure to sunlight, a parent molecule undergoes photoexcitation which induces its chemical transformation into a high energy photoisomer [8–13]. This photoisomer then can be stored for a certain period and thermal energy can be released when triggered with heat, light, catalyst, etc. The back isomerization of the metastable photoproduct regenerates the parent molecule for continuing the photoswitching cycle [8–13]. This enables the harvesting, conversion, and storage of solar energy into thermal energy via a reversible and closed photoswitching cycle.

The photoswitching couple must exhibit distinct features to be applicable for solar energy harvesting and storage [10]. These properties include the large storage energy, high gravimetric energy storage density, large barrier for thermal back isomerization reaction, photoabsorption in the visible range, high quantum yield of photoisomerization, and so forth [10]. Photoswitches like dihydroazulene/vinylheptafulvene, azobenzenes, tetracarbonyl(fulvalene)diruthenium complexes, norbornadiene/quadricyclane (NBD/QC), anthracenes, etc. exhibit a few promising properties for MOST applications [10,12–13]. However, these photoswitching couples still lack one or more important properties required in MOST systems and impose a serious limitation for their practical applications [8–13]. It is believed that the rational design of the molecular structure can assist to endow all the necessary properties in a single photoswitch [14]. Therefore, engineering of novel photochromic couples with an intention to incorporate all desired features within a single photoswitch is a focus of current research [15–30].

**Figure 1:**
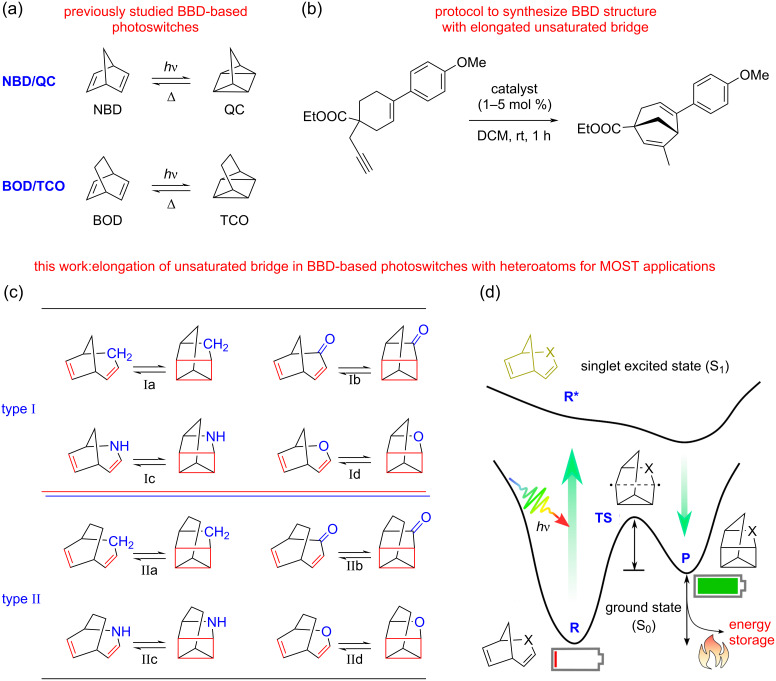
(a) Previously studied BBD-based photoswitches, (b) recently reported protocol to synthesize a BBD structure with elongated unsaturated bridge [37], (c) BBD-based photoswitches studied in the present work, (d) schematic representation of the photoswitching mechanism in the studied BBD-based photoswitches for molecular solar thermal energy storage applications.

The bridged bicyclic diene (BBD)-based NBD/QC couple involves a reversible [2 + 2]-cycloaddition and the chemical transformation during its photoswitching is depicted in Figure 1a. The NBD/QC couple is capable to chemically store huge amounts of solar energy due to the incorporation of large strain during the photoconversion and is thus recognized as a potential MOST candidate [8–10,13]. However, the optical absorption of unsubstituted NBD in the UV region hinders its practical applicability in MOST systems [8–10,13]. Several attempts have been made to red-shift the absorption wavelength in direction of the visible region by introducing different substitutions which often compromised the thermochemical properties [8–10,13]. Thus, the improvement of both the thermochemical and photophysical properties of a photoswitching couple is a challenging task.

The bicyclooctadiene/tetracyclooctane (BOD/TCO) couple structurally resembles the NBD/QC pair but has an extended saturated bridge due to the additional methylene (-CH_2_-) unit. An experimental report has evidenced that the BOD/TCO couple shows similar photoswitching behaviour (Figure 1a) and possesses a significantly better energy storage density, making it an interesting candidate for MOST applications [31]. The BOD molecule is likely to undergo a thermal degradation producing an aromatic ring via a kinetically controlled retro-Diels–Alder pathway [31]. However, the reversibility of the photoswitching cycle is a prerequisite for a closed MOST system. Recent studies have demonstrated that structural modifications in the basic BBD skeleton can alter the intrinsic properties of a photoswitch and may be crucial to impart novel photoswitching properties [31–36]. Further, the presence of heteroatoms in the bicyclic diene is also decisive to modulate the photoswitching behaviour [35–36]. This provides the impetus for rational designing of novel BBD photoswitches to endow all the essential functionalities in an individual system for a real-life MOST application.

Hitherto, studies have mainly focussed on the influence of saturated-bridge modifications on the photoswitching properties for applications in solar energy harvesting and storage [31–34]. A recent experimental study has reported a facile synthetic protocol (Figure 1b) to obtain BBD molecule which shows structural similarity with the NBD but has an elongated unsaturated bridge length [37]. Despite its potential, the photoswitching behaviour of this molecule has not been anticipated and the thermochemical and photophysical parameters relevant for MOST applications are still elusive.

Inspired by these earlier reports, herein we report our attempts to assess the impact of the unsaturated-bridge length elongation with different heteroatomic units on the thermochemical and photophysical properties of BBD-based photoswitches. The various BBD-based systems scrutinized in the present investigation are displayed in Figure 1c. These systems are rationally designed with elongated unsaturated bridge in both the NBD/QC and BOD/TCO pairs. Thus, these photoswitches can be basically classified into two types: (a) type-I is generated by increasing the unsaturated bridge length of the NBD/QC pair thus having a short saturated bridge, and (b) type-II designed by elongating the unsaturated bridge of the BOD/TCO pair thus have elongated saturated bridge, too. Further, the effect of the presence of different polarity solvents on the photoswitching properties has also been investigated for a real-life MOST application. The stability and the reversibility of the photoswitches have also been verified to attain a closed cyclic photoswitching process for practical MOST system.

## Results and Discussion

As can be seen from Figure 1a, the previously studied BBD-based photoswitches (NBD/QC and BOD/TCO) generate photoisomers with two strained 3-membered rings. In contrast, the photoinduced chemical transformation of all studied bicyclic dienes yield photoproducts containing one 4-membered and one 3-membered ring. Therefore, the metastable photoisomers of the studied photoswitches have a larger ring size and can be stabilized for longer time. Consequently, the TBR barrier can be enhanced which ultimately enables the harvested solar energy to be stored for an extended period of time. Further, elongating the unsaturated bridge with heteroatoms can assist to improve the photophysical properties. Thus, the studied photoswitches are rationally designed to incorporate both thermochemical and photophysical properties in a single photoswitching couple as desired in an ideal MOST system for solar energy harvesting and storage.

A schematic representation of the photoswitching mechanism operating in the studied BBD-based switches for MOST application is illustrated in Figure 1d. The parent BBD molecules absorb incoming photons from solar radiation and undergo photoinduced electronic excitation. The excited diene thereafter converts into a high-energy metastable photoproduct via a photoconversion process. The energy difference between the metastable photoproduct and the parent diene is the storage energy of the photoswitching pair and reflects the energy storage capacity of the MOST system. Thus, the larger energy difference between the two photoswitching isomers increases the amount of energy harnessed from solar radiation. Owing to its high energy, the metastable photoisomer shows a propensity to revert back to the parent diene and heat is released during this process. The regenerated parent diene further continues the photoswitching cycle. The activation energy required to trigger the transformation of the metastable photoisomer to the parent diene is called the thermal back isomerization barrier. The TBR barrier governs the duration of storage of harvested solar energy in the MOST devices. Photoswitching systems exhibiting a large TBR barrier store solar energy for long periods of time.

### Thermochemical properties

**Figure 2:**
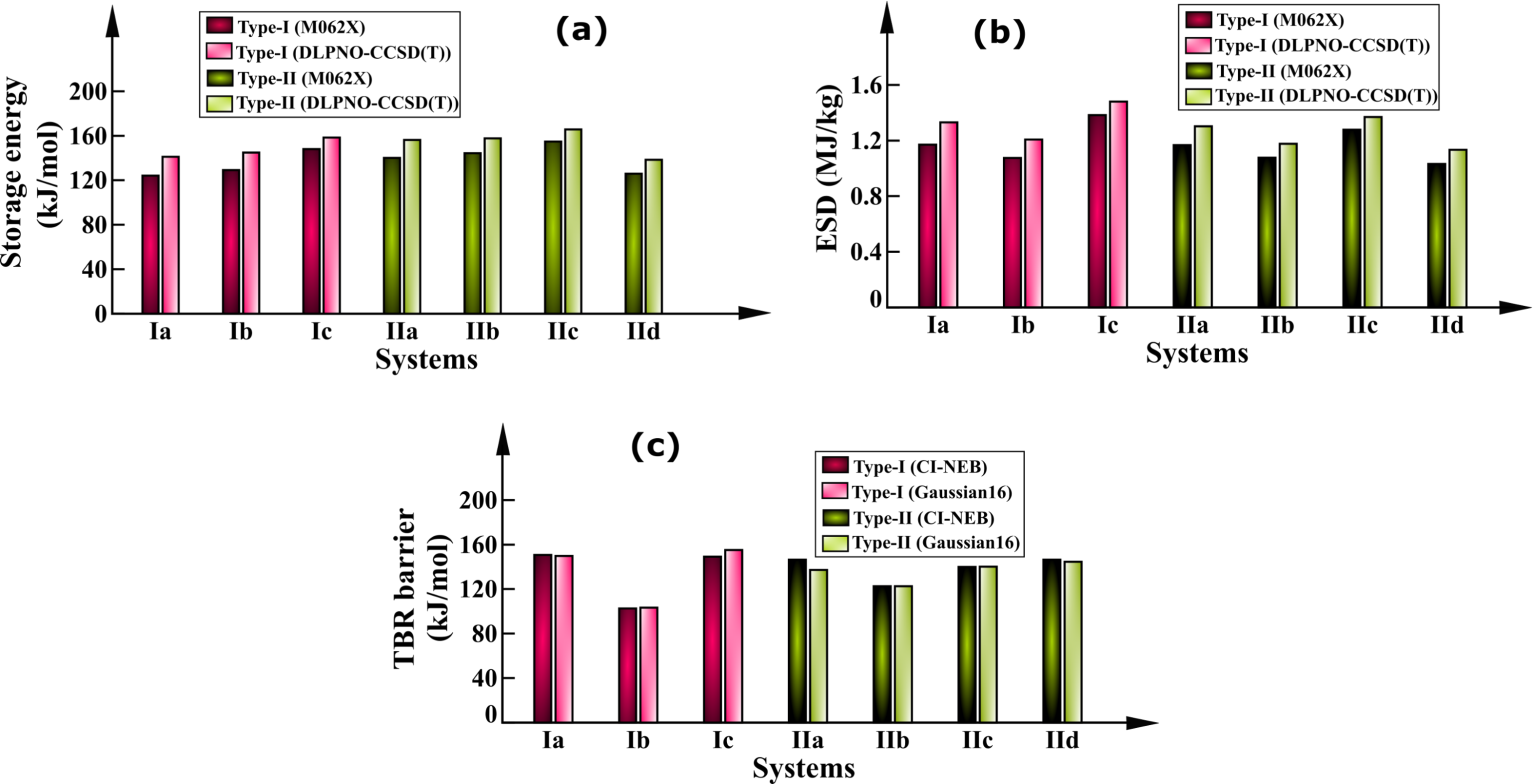
Energy storage capacities and barrier for back conversion of the photoproduct to the diene in the gas phase. (a) Storage energy, (b) energy storage density, (c) TBR barrier.

En route to probe the energy storage capacity of the studied BBD-based photoswitches, initially the storage energy and energy storage density (ESD) have been calculated. Figure 2 displays the energy storage capacity of the studied photoswitches calculated employing M062X/6-311++G** and DLPNO-CCSD(T)/Def2TZVP level. The calculated storage energy and energy density values are provided in Table 1.

**Table 1:** Thermochemical properties of the studied BBD-based photoswitches and previously reported NBD/QC and BOD/TCO pairs in the gas phase.^a^

Systems	Storage energy (kJ/mol)		ESD (MJ/kg)		TBR barrier (kJ/mol)	

	M062X	DLPNO-CCSD(T)	M062X	DLPNO-CCSD(T)	CI-NEB	Gaussian16

Ia	124.14	141.15	1.17	1.33	150.71	149.64
Ib	129.11	144.91	1.08	1.21	102.56	103.37
Ic	148.08	158.38	1.38	1.48	149.23	155.19
IIa	140.08	156.35	1.17	1.30	146.49	137.14
IIb	144.38	157.66	1.08	1.18	122.57	122.59
IIc	154.83	165.74	1.28	1.37	139.85	140.02
IId	125.93	138.28	1.03	1.13	146.43	144.58
NBD/QC	96.30		1.05		124.10	
BOD/TCO	192.80		1.82		118.10	

^a^The thermochemical properties of the NBD/QC and BOD/TCO pairs are taken from the previous study where energy storage capacities were calculated with CPS(D-4) for geometries optimized with M06-2X/6-311++G** and TBR barriers were accounted at the (8,8)-CASPT2/6-311++G** level for the TS obtained using the NEB [33].

The storage energies calculated using the DLPNO-CCSD(T) level are in the range of 141.15 to 158.38 kJ/mol for the type-I switches and 138.28 to 165.74 kJ/mol for the type-II photoswitches. It is worth to mention that the storage energy of the studied BBD-based switches is significantly higher than that of the prototype NBD/QC pair (96.06 kJ/mol) [36]. It is also apparent from Figure 2 that type-II photoswitches which are based on the BOD/TCO pair have a higher storage energy than the type-I systems which are based on the NBD/QC couple. These results are consistent with a previous report which demonstrated that the energy storage capacity of the BOD/TCO system (192.80 kJ/mol) is higher than that of the NBD/QC couple (96.30 kJ/mol) [33]. Moreover, the energy storage density ranges from 1.21 to 1.48 MJ/kg for type-I switches and 1.13 to 1.37 MJ/kg for type-II switches. The gravimetric energy densities of the studied photoswitches is higher than that of the NBD/QC pair (1.04 MJ/kg) [36]. Therefore, the elongation of the unsaturated bridge results in a higher energy storage capacity compared to the unsubstituted NBD/QC photoswitch.

It has also been observed that M062X/6-311++G** considerably underestimates the energy storage capacity of the studied BBD-based photoswitches when compared with the DLPNO-CCSD(T)/Def2TZVP level (Table 1). Also, the energy storage capacity of type-Ia, Ib, Ic and IIa, IIb, and IIc systems is the highest among all the studied photoswitches. This infers that –CH_2_–, –(CO)–, and –NH– are better elongation units of the studied BBD-based photoswitches for improving energy storage capacities.

The TBR barrier plays a decisive role in determining the energy storage time of the photoswitching system. The singlet biradicaloid TS involved during the thermal back isomerization of photoproduct to the parent bicyclic diene were initially predicted with the PBE functional employing CI-NEB method analogous to the previous studies [33,36]. The obtained TSs were re-optimized with PBE/6-311++G** level utilizing the Gaussian16 and vibrational analysis was performed at the same level of theory. The TS was confirmed as a first-order saddle point by the presence of a single imaginary frequency whose displacement vectors are in the direction of bond forming and breaking. The optimized geometry of the TSs along with the displacement vectors in the direction of bond forming and breaking is provided in Figure S1 (Supporting Information File 1).

The TBR barrier estimated using the (8,8)-CASPT2/6-311++G** method for the TS geometry obtained using CI-NEB and Gaussian16 is shown by histograms in Figure 2c. These TBR barriers calculated using both methods are also provided in Table 1. It is clear from Figure 2c that the TBR barrier solely depends on the type of heteroatom unit used to extend the unsaturated bridge length of the bicyclic diene. The TBR barrier is low only for type-Ib (103.37 kJ/mol) and IIb (122.59 kJ/mol) systems, while the other studied photoswitches have higher TBR barriers (137.14 to 155.19 kJ/mol). Noteworthy, these TBR barriers are significantly higher than those of the unsubstituted NBD/QC (124.10 kJ/mol) and BOD/TCO (118.10 kJ/mol) couples [33]. This is indeed expected due to the formation of comparatively stable and large rings in the metastable photoproducts. Among all the studied BBD switches, the barrier height for back isomerization of the photoproduct to the diene is largest for systems whose unsaturated bridge was extended with –CH_2_–, –NH–, and –O– units. Interestingly, these TBR barriers for the studied BBD-based photoswitches with elongated unsaturated bridges are substantially higher and can store the energy for several months and even longer.

Overall, the studied BBD-based photoswitches with elongated unsaturated bridges have higher energy storage capacities than the NBD/QC pair. The storage energy is found to be higher for systems whose unsaturated bridge lengths are extended with –CH_2_–, –(CO)– and –NH– units. Additionally, extension of the unsaturated bridge of the BBD molecule relieves strain in the metastable photoisomer and thus, enhances the TBR barrier. The calculated energy barrier of TBR was found to be largest for the systems whose unsaturated bridge was extended with –CH_2_–, –NH–, and –O– units. Therefore, it is worth to mention that elongating the unsaturated bridge length of BBD molecule improves the thermochemical properties. As a result, the studied BBD-based photoswitches show the potential for efficiently harvesting solar radiation and long-term storage of energy in the chemical bonds of the photoproduct. More particularly, the BBD-systems whose unsaturated bridge was extended using –CH_2_– and –NH– units exhibit the best storage energy capacities as well as the highest TBR barriers.

**Figure 3:**
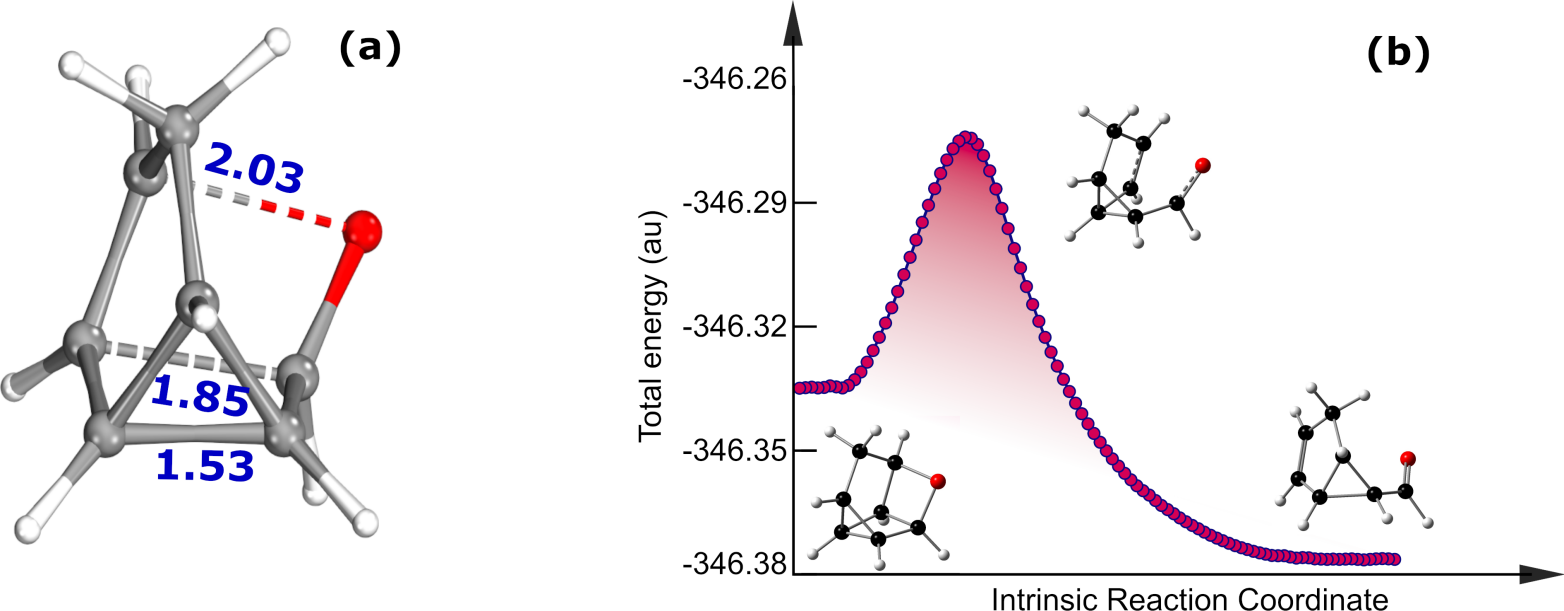
(a) Optimized geometry along with the important geometrical parameters of the TS obtained for the thermal degradation of the photoisomer of the Id system and (b) the IRC profile for the conversion of photoisomer of the type-Id system to undesired by-product.

It can be noted that we were unable to find the TS for the thermal back conversion of the type-Id system by employing CI-NEB and Gaussian 16. Several attempts to search the TS structure instead resulted in a distinct TS geometry than that involved in thermal back conversion. The optimized geometry of the TS for the Id system along with the important geometrical parameters are shown in Figure 3. Analysis of the geometrical parameters reveals that the TS is associated with dissociation of the photoproduct. To validate the interconnection of the TS with the metastable photoisomer and the expected degradation product, IRC calculations were performed. The IRC calculations confirm that the obtained TS interlinks the metastable photoproduct with an undesired byproduct (Figure 3).

Calculations reveal that the barrier for thermal dissociation of the photoproduct type-Id (209.43 kJ/mol) is significantly larger than the TBR barrier accounted for all the studied photoswitches. Nevertheless, the results reflect the propensity of the type-Id system to yield an undesired byproduct due to thermal dissociation. Consequently, the type-Id switch does not meet the criteria for reversible photoswitching desired for an ideal MOST system. This leads to the inference that the heteroatom used to extend the unsaturated bridge of the BBD molecules plays a crucial role in determining the stability of the metastable photoproduct against undesired thermal degradation.

### Photophysical properties

**Figure 4:**
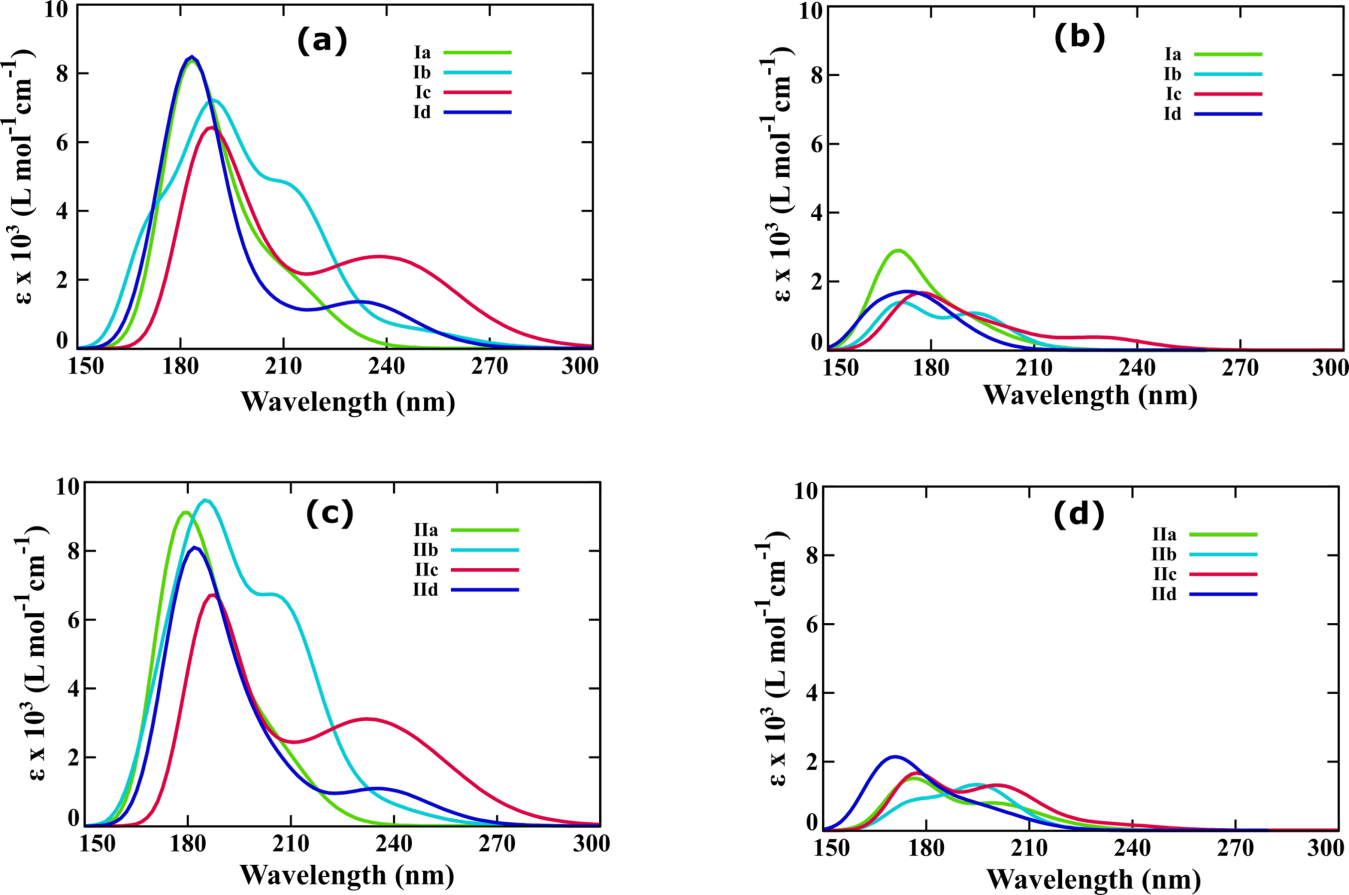
Photophysical properties of the studied BBD photoswitches with elongated unsaturated bridges. (a) Optical spectra of dienes of type-I switches and (b) optical spectra of photoproducts of type-I photoswitches, (c) optical spectra of dienes of type-II photoswitches, and (d) optical spectra of photoproducts of type-II switches.

Photoabsorption of bicyclic dienes in the visible region of the solar spectrum is one of the prerequisites for their applicability as photoswitches in MOST systems. Therefore, forecasting the photophysical properties of BBD switches is one of the prominent objectives of the present investigation. The optical spectra of the dienes and photoproducts of both the types of BBD switches are depicted in Figure 4.

The optical spectra were obtained as the outcome of the first fifteen singlet vertical electronic excited state energy values computed with the TD-M062X/6-311++G** utilizing Gaussian 16. The data was extracted by considering the half width of 0.33 at the half height of the optical spectrum. The calculated spectral data including the absorption onset (λ_onset_), the first important electronic excitation wavelength (λ) whose oscillatory strength (*f*) surpasses 0.01, and the molar absorption coefficient (ε) are systematically tabulated in Table 2. The results are compared with the optical properties previously reported for the NBD/QC and BOD/TCO pairs computed at the resolution-of-identity-CC2 model in conjunction with the 6-311++G** basis set [33].

**Table 2:** Spectral data for dienes and photoproducts of the studied BBD-based photoswitches calculated with M062X/6-311++G** level of theory and previously reported NBD/QC and BOD/TCO pairs.^a^

Systems	λ_onset_	*f*	λ (nm)	ε (L mol^−1^ cm^−1^)	λ_onset_	*f*	λ (nm)	ε (L mol^−1^ cm^−1^)

	diene				photoproduct			

Ia	241.12	0.029	212.80	2151.00	216.48	0.010	185.09	1377.58
Ib	275.00	0.013	247.18	602.84	215.60	0.014	195.18	1027.32
Ic	295.60	0.043	249.65	2375.97	251.20	0.011	177.38	1669.30
Id	268.50	0.021	238.21	1266.49	205.28	0.013	177.10	1638.78
IIa	233.92	0.022	206.58	2697.24	229.96	0.010	172.85	1376.49
IIb	261.50	0.013	235.86	877.54	220.40	0.013	198.93	1245.59
IIc	292.20	0.029	251.62	2162.12	247.40	0.013	202.79	1292.60
IId	269.50	0.015	240.38	1028.23	220.24	0.010	194.97	791.19
NBD/QC	–	0.017	213.65	–	–	0.019	190.74	–
BOD/TCO	–	0.041	196.44	–	–	0.027	204.92	–

^a^The spectral data of NBD/QC and BOD/TCO pairs was previously reported at the resolution-of-identity-CC2 model in conjunction with 6-311+ +G** basis set [33].

From the figure, it can be clearly seen that the molar absorption coefficient of the parent dienes is significantly larger when compared with those of the metastable photoproducts. Close analysis of the optical spectra unraveled that the unit used to elongate the unsaturated bridge of the BBD-based photoswitch plays a critical role in altering the photophysical properties. The calculated first important electronic excitation wavelength and the absorption onset are longest for the diene whose unsaturated bridge length was extended using –(CO)– and –NH– units. The calculated λ (λ_onset_) for the dienes of type-Ib, Ic, IIb, and IIc photoswitches are 247.18 (275.00), 249.65 (295.60), 235.86 (261.50), and 251.62 (292.20) nm, respectively (Table 2). These absorption wavelengths are significantly longer than the previously reported λ for NBD (213.65 nm), BOD (196.44 nm), QC (190.74 nm), and TCO (204.92 nm) calculated at the resolution-of-identity-CC2 model in conjunction with the 6-311+ +G** basis set [33]. Also, the calculated λ for the photoproducts of type-Ib, Ic, IIb, and IIc photoswitches are 195.18, 177.38, 198.93, and 202.79 nm, respectively (Table 2). Therefore, the optical absorption spectra unveil that the extension of the unsaturated bridge length with –(CO)– and –NH– units as in type Ib, Ic, IIb, and IIc photoswitches red-shifts the excitation wavelength of the bicyclic diene and helps improving the photophysical properties.

More importantly, the differences between the first important excitation wavelength of the diene and photoproduct are 52 and 72 nm for the type-Ib and Ic photoswitches and are larger than that of the NBD/QC pair (30 nm) [36]. This implies that the spectral overlap between the diene and the photoproduct is slightly reduced for the type-I photoswitches whose unsaturated bridge were extended using –(CO)– and –NH– units. This will assist to avoid achieving photostationary state (PSS) during the optical excitation of the diene. Therefore, elongation of an unsaturated bridge using a single unit has the potential to improve the photophysical properties, particularly the absorption cross-section, excitation wavelength, and spectral overlap. Despite this, the parent dienes of the studied BBD-based photoswitches show absorption in the UV region of the electromagnetic spectrum. Harvesting solar energy reaching the Earth’s surface requires efforts to be devoted to red-shift the absorption wavelengths of the bicyclic diene in the visible range. As previous reports have demonstrated, this can be practically achieved by substitution with electron-releasing or accepting groups [8–9,33].

The energy storage capacity of the photoswitches should always be less than the actual solar energy harnessed by the bicyclic molecule. To perform this reality check, the photon energy (*E*_photon_) for the first important electronic excitation (whose oscillatory strength is higher than 0.01) was compared with the sum of the storage energy and TBR barrier (SE + TBR barrier) for the studied photoswitches. The calculated values are provided in Table S1 of Supporting Information File 1. The sum of storage energy and TBR barrier is always less than the photon energy (SE + TBR barrier) < *E*_photon_) involved in the first important electronic excitation. This validates the reality of the photoswitches and reliability of the obtained outcomes. The solar energy conversion efficiencies (η_efficiency_) of the studied photoswitches have also been estimated as demonstrated in a recent report [34]. The calculated results are also provided in Table S1 of Supporting Information File 1 and are within the range of 7.78 to 10.31% for the studied BBD-based photoswitches. These η_efficiency_ values are in agreement with the solar energy conversion efficiencies of the BBD-based photoswitches with modified saturated bridges [34].

### Effect of solvation

Knowledge of the best suitable environmental conditions for the photoswitching system is essential to achieve high performance in the real-life MOST devices. Therefore, attention has also been devoted to envisage the impact of different polarity solvents on the properties of the BBD-based photoswitching couples. Herein, the influence of solvation has been measured only for type-Ia, Ic, IIa, and IIc photoswitching couples. Initially, the geometries of the bicyclic dienes and photoproducts of the studied photoswitches were optimized in the presence of different polarity solvents at the M062X/6-311++G** level considering the SMD model. Thereafter, the storage energies were computed by performing the single point energy calculations at DLPNO-CCSD(T)/Def2TZVP and including the thermal correction at the M062X/6-311++G** level. Likewise, the geometries of the TS were optimized with PBE/6-311++G** in presence of different polarity solvents considering SMD model and TBR barrier were accounted at the (8,8)-CASPT2/6-311++G** level.

**Figure 5:**
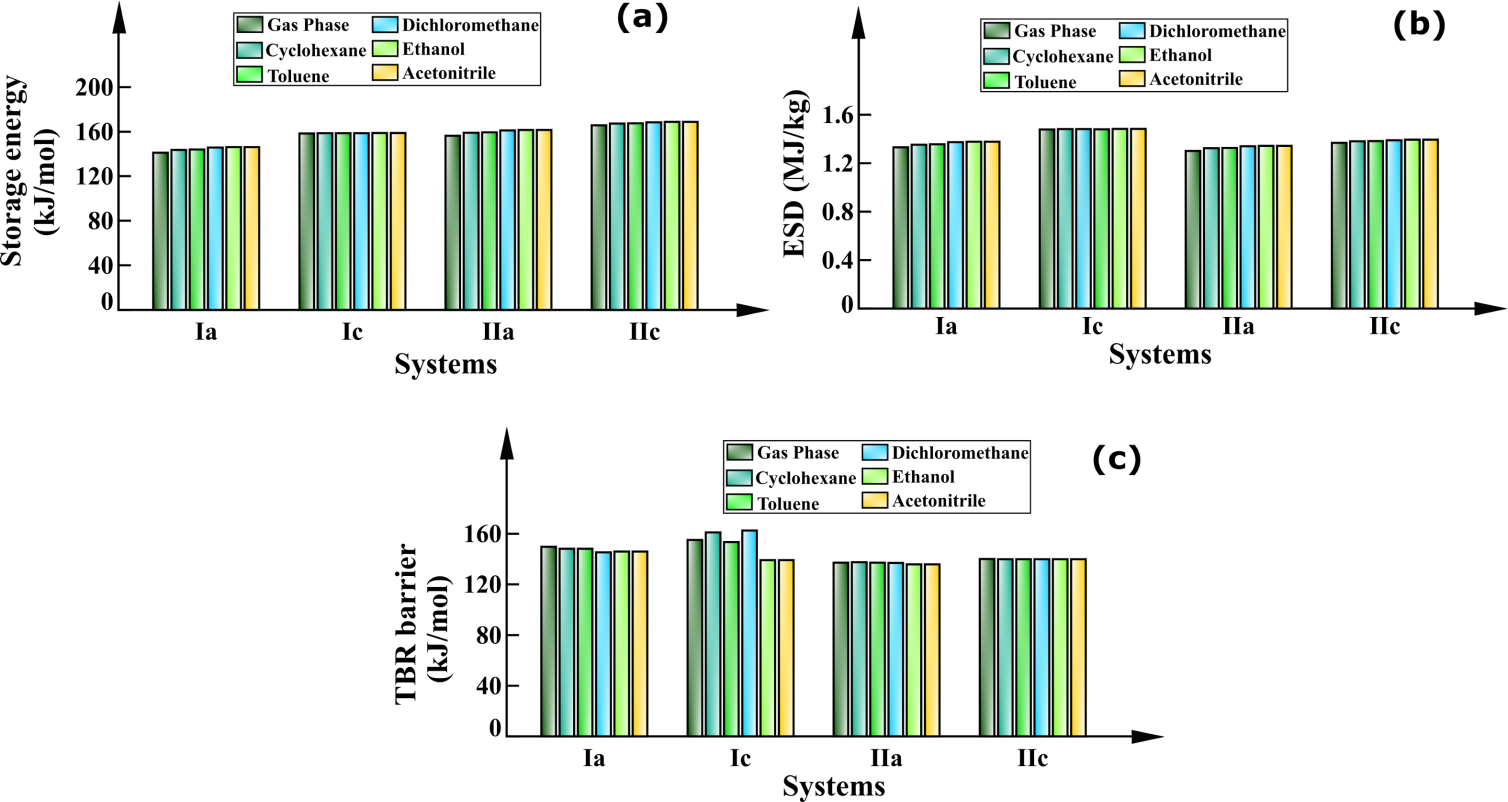
Effect of solvation on the thermochemical properties of the studied BBD photoswitches considering the SMD solvation model. (a) Storage energy, (b) energy storage density calculated at DLPNO-CCSD(T)/Def2TZVP level, and (c) TBR barrier accounted at (8,8)-CASPT2/6-311++G** level.

The effect of different polarity solvents on the thermochemical properties of the photoswitching couples considering the SMD solvation model is illustrated with histograms in Figure 5. The estimated data for storage energy, energy storage density, and TBR barriers in the presence of different solvents are given in Table S2 of Supporting Information File 1.

As can be seen from Figure 5 the storage energy and energy storage density appreciably increase due to solvation of the photoswitches in the different polarity solvents considering the SMD solvation model. The increase in energy storage capacity becomes more pronounced with increasing polarity of the solvent. The extent of increment in storage energy upon solvation is largest for the type-Ia (2.42–5.07 kJ/mol) and IIa (2.60–5.20 kJ/mol) photoswitching couples while the variation is least for the Ic (0.18–0.43 kJ/mol) switches. The trend observed for the effect of solvation on energy storage capacity is in agreement with a recent investigation on solvated BOD/TCO systems [38]. Juxtaposition to this, mostly the TBR barrier considerably decreases as the polarity of the solvent increases. The largest decrease in the TBR barrier is noticed for the type-Ia (1.37–4.38 kJ/mol) and Ic (−1.75–18.83 kJ/mol) photoswitches whereas the decrease in TBR for the type-IIc (0.19–0.32 kJ/mol) photoswitch is only marginal. Additionally, a little enhancement in the barrier for the thermal back isomerization is noticed for the type-IIa (0.30–0.36 kJ/mol) photoswitch with increasing polarity of the solvent. Due to the large convergence issue for the solvated systems of the type-IIc photoswitch, the electronic energies with (8,8)-CASPT2/6-311++G** were calculated with loose SCF criterion and may attribute to the large deviation between the TBR barriers in different polarity solvents (Figure 5). The result signifies that the energy storage capacity of the type-Ia and Ic photoswitches can be improved with increasing polarity of solvents but at the cost of the TBR barrier. Further, the high polarity solvents can improve the energy storage capacity of the photoswitches without compromising the TBR barrier for type-IIa and IIc photoswitches.

To probe the effect of solvation model, the calculations were performed by considering the polarizable continuum model (PCM) solvation model and systematically compared with the SMD model. The influence is assessed by evaluating the storage energies of the type-Ia and Ic photoswitches in different polarity solvents using both the solvation models at the M062X/6-311++G** level of theory. The calculated energy values are displayed by histograms in Figure S2 of Supporting Information File 1. The trend observed for the storage energies of the photoswitches in different polarity solvents calculated with M062X/6-311++G** (PCM solvation model) closely resembles with those obtained with DLPNO-CCSD(T) (SMD model). However, the storage energies of the photoswitches in different polarity solvents calculated at the M062X/6-311++G** level considering the SMD model are lower than the PCM model. Since the effect of solvation model is assessed at the M062X/6-311++G** level, the computed energy values are different than those obtained with the DLPNO-CCSD(T) level of theory. However, the influence of the solvation model on the overall storage energies in presence of different polarity solvents is only marginal.

**Figure 6:**
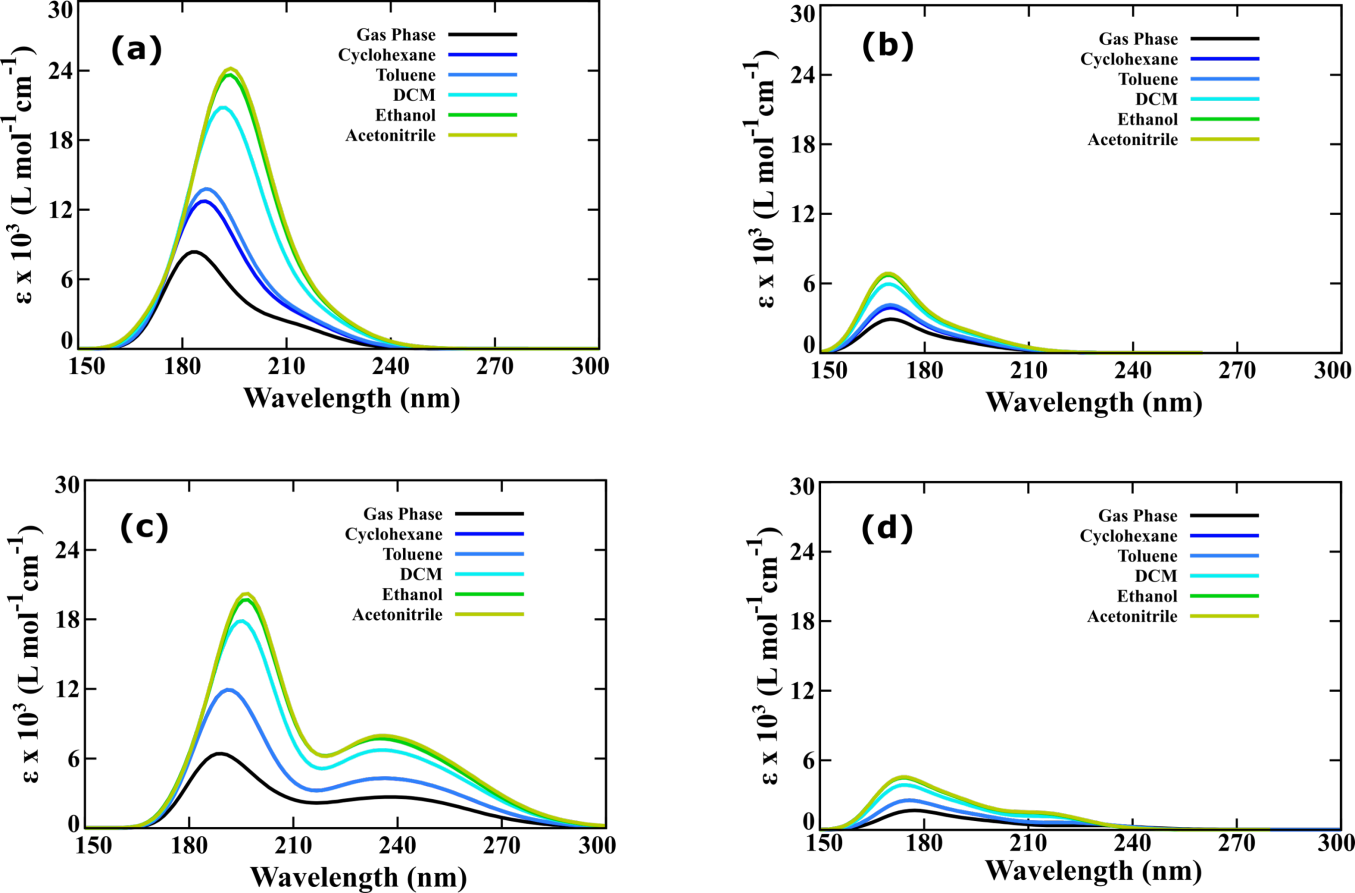
Effect of solvation on the photophysical properties of the studied BBD photoswitches. (a) Optical spectra of diene of type-Ia photoswitch, (b) optical spectra of photoproduct of type-Ia photoswitch, (c) optical spectra of diene of type-Ic photoswitch, and (d) optical spectra of photoproduct of type-Ic photoswitch (M062X/6-311++G**).

Furthermore, the influence of different polarity solvents on the photophysical properties of the parent dienes and the metastable photoproducts has also been forecasted. The optical absorption spectra for the solvated dienes and photoproducts of type-Ia, and Ic photoswitches are displayed in Figure 6. The calculated absorption spectra for the solvated diene and photoproducts of type-IIa and IIc photoswitches are provided in Figure S3 of Supporting Information File 1. The calculated spectral data in the presence of different solvents are systematically tabulated in Table S3 and Table S4 of Supporting Information File 1.

Figure 6 clearly elucidates that the absorption intensity of the diene and photoproduct remarkably enhances with solvation. The molar absorption coefficient is maximum for the highest polar solvent. Additionally, the absorption cross section is largest in the presence of the highly polar solvent, acetonitrile (dielectric constant = 35.688). The increase in absorption intensity is more pronounced for the diene when compared to the metastable photoproduct. Therefore, the difference in the absorption coefficients of the diene and corresponding photoproduct becomes larger with increasing polarity of the solvent. It is interesting to note that a considerable bathochromic shift in the excitation wavelength is also noticed with increasing polarity of the solvents. The red-shift of 7.56 to 12.30 nm is observed in the presence of highly polar acetonitrile. Noteworthy, the absorption onset of the bicyclic diene of type-Ic and IIc is red-shifted to 305 and 304 nm, respectively, in the presence of acetonitrile. This evidences that the solvation of the photoswitches with highly polar solvents improves their photophysical properties.

### Thermal degradation of photoisomers

The reversibility of the photoswitching process is one of the key requisites for the applications in molecular solar thermal energy storage. Therefore, the feasibility of the thermally reversible photoswitching in the studied BBD-based systems has also been assessed. Initially, the relative energy profiles for the conversion of the photoproducts to the dienes and undesired thermal degradation products were generated. The thermal stability and reversibility of the photoswitching cycle of the type-IIa photoswitch was analyzed using the ab initio molecular dynamics (AIMD) simulations.

**Figure 7:**
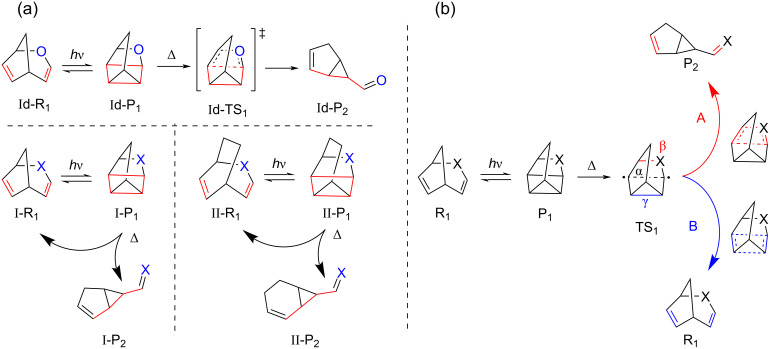
The undesired degradation competing the thermal back conversion of the photoproduct. (a) The thermal degradation of the photoproduct leading to undesired by-products competing with the thermal back isomerization and (b) mechanism of thermal degradation and back isomerization reaction of the metastable photoproduct.

The proposed mechanism for the thermal back conversion and undesired thermal degradation of the photoproduct is illustrated in Figure 7. As mentioned earlier, the dissociation of the two newly formed σ-bonds proceeds in a highly asynchronous mechanism. Therefore, initially, only one of the two σ-bonds denoted as α bond in the photoproducts (Figure 7) dissociates to form a TS structure having singlet biradicaloid. Subsequently, there exists a competition for the dissociation of the β or γ-bond that yields undesired thermal degradation products through pathway A or the parent diene via pathway B, respectively. This dissociation follows a highly asynchronous but concerted reaction mechanism and may involve bispericyclic post-transition state bifurcation (PTSB) of the PES [39–40]. Consequently, the reaction tends to produce two different products from a single TS. Thus, the reaction resembles a peculiar ambimodal reaction that can produce multiple products from a single TS structure [41].

**Figure 8:**
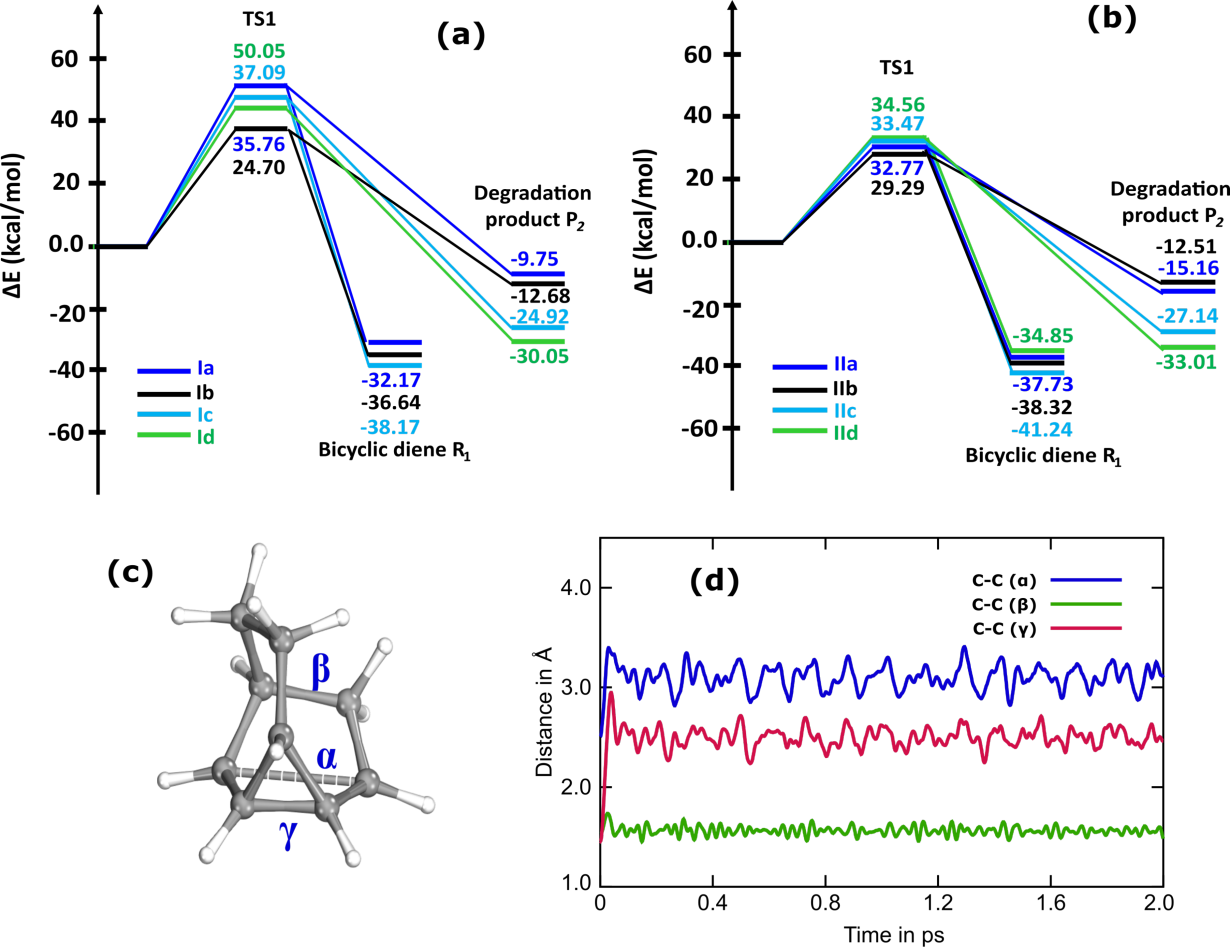
Relative energy profiles for the conversion of the photoproducts into the parent dienes and undesired degradation products under thermal conditions. (a) Relative energy profile for type-I photoswitches, (b) relative energy profile for type-II photoswitches ((8,8)-CASPT2/6-311++G**), (c) singlet biradicaloid TS representing the bond distances sampled during the AIMD simulation, and (d) variation in important bond distances during the course of the AIMD simulations.

Owing to the formation of two singlet biradicaloids in the TS structure, the single reference DFT functionals are inappropriate to describe the TS. Despite this, the previous benchmark study has demonstrated that the PBE functional is still superior for predicting the geometry of the TS [42]. Therefore, the geometry of TS structures was herein determined using the PBE/6-311++G** method utilizing the Gaussian 16. Thereafter, the relative energy profiles were generated by computing the electronic energies of the point structures at the (8,8)-CASPT2/6-311++G** level of theory. Attempts to find the TS (both with CI-NEB and Gaussian 16) for the thermal back isomerization of the photoproduct of type-Id photoswitch to the corresponding diene have resulted in a TS that is associated with undesired thermal degradation (Figure 3). The obtained TS follows a conventional, synchronous and concerted dissociation mechanism to produce the undesired carbonyl compound (Id-P_2_) shown in Figure 7. Cartesian coordinates of all the optimized geometries are provided in Supporting Information File 1. Figure 8 depicts the calculated relative energy profiles for the reversible thermal back conversion of the photoproduct to the bicyclic diene and the undesired thermal degradation of the photoproduct.

It is evident from the energy profiles that the parent dienes (R_1_) obtained by thermal back isomerization of the photoproducts (P_1_) are energetically more stable when compared with the thermally degraded products (P_2_). Since a single TS is involved in regenerating the parent diene and producing the undesired product resembling the ambimodal reaction, the reversible photoswitching is more probable due to the energetic stability of the parent diene. Further, it has been noticed that the energetic stability of the undesired product depends on the unit used to extend the unsaturated bridge of the bicyclic diene. The energetic stability of the undesired byproduct is lowest for the photoswitching system whose bridge length is elongated with –(CO)– and –CH_2_– units followed by –NH– and –O– units.

The calculated energy difference between the parent diene and the competing byproduct is largest for Ia (22.42 kcal/mol), IIa (22.57 kcal/mol), Ib (23.96 kcal/mol), IIb (23.80 kcal/mol) photoswitches. On the other hand, the energy difference between the parent diene and competing byproduct is least for the Id (5.76 kcal/mol) and IId (1.84 kcal/mol) photoswitches. This increases the propensity of the Id and IId couples towards the undesired thermal degradation product. This clearly indicates that the unit used for elongation of the unsaturated bridge is crucial to control the reversibility of the photoswitching cycle or the stability of the photoproduct against undesired thermal degradation. Among the studied BBD-based system, the metastable photoproducts of the photoswitching couple whose unsaturated bridge was extended using the –O– (Id and IId, respectively) are prone to undergo undesired thermal degradation. In contrast, the metastable photoproducts of the photoswitching couple whose unsaturated bridge was extended using the –(CO)–, –CH_2_–, and –NH– show thermodynamic feasibility for reversible photoswitching.

Furthermore, the thermal reversibility of the photoswitching process has been validated by AIMD simulations initiated using the biradicaloid TS structure of the IIa photoswitch. The variation in α, β, and γ-bond distances calculated during the AIMD dynamic trajectory is displayed in Figure 8. It is apparent from the figure that the α and γ-bond distances increase during the course of the simulations whereas the β distance almost remained constant. This signifies that subsequent formation of the TS, reversible photoswitching is more feasible over the thermal degradation of the metastable photoproduct. The outcomes of the AIMD simulations can be clearly visualized in a movie provided in Supporting Information File 2.

## Conclusion

The outcomes of the simulations advocate that the heteroatomic unit used to elongate the unsaturated bridge is crucial in determining the stability, reversibility, and photoswitching properties of bicyclic dienes. DLPNO-CCSD(T) and (8,8)-CASPT2 results reveals that the photoswitches whose unsaturated bridge was extended using –CH_2_– and –NH– units exhibit the best storage energy capacities and high TBR barriers. The calculated energy storage capacities (138.28–165.74 kJ/mol) of the studied bridged bicyclic dienes are ≈1.5 times higher than the prototype NBD/QC (≈96 kJ/mol) pair in the gas phase. The estimated energy barriers for the thermal back isomerization in the studied photoswitching systems are large enough to store the harvested energy for several months or longer in the gas phase. The time-dependent calculations using the TD-M062X functional reveal that an elongation of the unsaturated bridge using –(CO)– and –NH– red-shifts the absorption onset and excitation wavelength and thus are the best elongation units to improve the photophysical properties. The energy storage capacity of the type-Ia and Ic photoswitches increases as the polarity of solvent goes on increasing (0.18–5.07 kJ/mol) while the TBR barrier considerably decreases (1.36–18.83 kJ/mol). Surprisingly, the energy storage capacity of the type-IIa (5.20 kJ/mol) and IIc (3.14 kJ/mol) photoswitches appreciably increases with the polarity of the solvent without compromising the TBR barrier. The solvation of studied photoswitching systems displays a bathochromic shift in the absorption onset and excitation wavelength and remarkably enhances the absorption intensities. The effect of solvation is more pronounced with the increasing polarity of the solvents. The photoswitching systems may show propensity for the undesired thermal degradation of the photoisomer via an ambimodal pathway, however the energetic stability of the parent diene favours the reversible photoswitching. Extending the unsaturated bridge of the bicyclic diene with an –O– unit promotes the thermal degradation of the photoproduct to the carbonyl compounds. Among the studied systems, the bicyclic diene photoswitch whose unsaturated bridge length has been extended using a –NH– unit exhibits the best storage energy (158.57 kJ/mol), energy storage density (1.48 MJ/kg), TBR barrier (136.36 kJ/mol), and absorption onset (305.00 nm) in the presence of acetonitrile. These values are substantially higher when compared with the storage energy (96.06 kJ/mol), energy storage density (1.04 MJ/kg), and TBR barrier (121.76 kJ/mol) of the prototype NBD/QC pair in the gas phase. This work provides a new avenue to design bridged bicyclic dienes to achieve improved photoswitching properties in a single system and the results will certainly have a remarkable impact for applications in solar energy harvesting and storage.

## Computational Details

The molecular structures of all studied dienes and photoproducts were fully relaxed without any geometrical or symmetrical constraints. The geometry optimization was performed using the M062X functional in combination with the 6-311++G** Pople-type basis set. This method was employed since the previous benchmarking studies have reported that the M062X/6-311++G** level performs well for prediction of the geometry and photoswitching properties of the BBD based systems. The analytical second order derivatives were also calculated to characterize the nature of the point structures. The critical point structure with no imaginary frequency was confirmed as a local minimum on the potential energy surface (PES). These optimized geometries were utilized for the further calculations including single point electronic energy, thermochemical and photophysical properties, etc.

The photophysical properties of the dienes and photoproducts were determined using the time-dependent TD-M062X/6-311++G** level of theory. The calculations were performed to determine the vertical excitation energy and oscillatory strength for the first fifteen singlet electronic excited states. To assess the influence of solvents, the geometries were optimized and photoswitching properties were determined in different polarity solvents (dielectric constant) including cyclohexane (2.0165), toluene (2.3741), dichloromethane or DCM (8.93), ethanol (24.852), and acetonitrile (35.688). Initially, the geometries of the bicyclic dienes and photoproducts of the studied photoswitches were optimized in the presence of different polarity solvents at the M062X/6-311++G** level considering the SMD model. Thereafter, the storage energies were computed by performing the single point energy calculations at DLPNO-CCSD(T)/Def2TZVP and including the thermal correction at the M062X/6-311++G** level. Likewise, the geometries of the TS were optimized with PBE/6-311++G** in presence of different polarity solvents considering SMD model and TBR barrier were accounted at the (8,8)-CASPT2/6-311++G** level. These calculations performed in the presence of solvents were modelled using the continuum solvation model density (SMD) solvation model. The pure implicit SMD solvation model has poor reliability to predict the p*K*_a_ values, and therefore, the storage energy was also calculated using the polarizable continuum model (PCM) [43]. The storage energy calculated using both the methods are systematically compared. All the above-mentioned calculations were accomplished using the density functional theory (DFT) implemented in Gaussian 16 [44].

The storage energy (Δ*G*_str_) is the amount of harvested solar energy that can be converted and stored as chemical energy in the metastable photoisomer. It measures the energy storage capability and the amount of thermal energy that can be obtained from a photoswitch in a MOST system. It can be calculated employing the following equation:


[1] $$\Delta G_{\text{str}}=G_{\text{photoproduct}}-G_{\text{diene}},$$

where *G*_diene_ and *G*_photoproduct_ indicate the Gibbs free energy of the bridged bicyclic diene molecule and the corresponding photoproduct. The Δ*G*_str_ was herein calculated by computing the Gibbs free energy of the diene and photoproduct using the M062X/6-311++G** level for the geometries optimized using the same level of theory. Further, the single point energy of the diene and photoproduct were also accounted at the DLPNO-CCSD(T)/Def2TZVP level of theory to predict the more accurate storage energy values at a low computational cost. This storage energy is herein designated as 
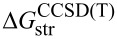
 and was estimated by including the thermal correction at M062X/6-311++G** level to the electronic energies computed at the DLPNO-CCSD(T)/Def2TZVP for the geometries optimized with M062X/6-311++G**.


[2] $$\Delta G_{\text{str}}^{\text{CCSD(T)}}=\Delta E_{\text{str}}^{\text{CCSD(T)}}+\Delta T,$$

where 
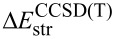
 is the difference in electronic energies (DLPNO-CCSD(T)/Def2TZVP) of the diene and photoproduct and Δ*T* is the difference in thermal contribution to the Gibbs free energy (M062X/6-311++G**) of the two photoisomers.

The thermal back reaction (TBR) barrier is the energy barrier that the photoproduct must overcome to revert back into the parent isomer for continuing the photoswitching cycle. The dissociation of two newly formed σ-bonds during the thermal back isomerization proceeds in a highly asynchronous mechanism. Initially, only one of the two newly formed σ-bonds is dissociated leading to the formation of singlet biradicals in the transition state (TS) structure. Therefore, the TS involved in the back reversion of the metastable photoisomer to the parent molecule exhibits multireference character. The single reference DFT functionals are unable to properly describe this multiconfigurational behaviour and often overestimate the TBR barrier. Therefore, the multireference complete active space (CAS) methods like CASPT2 are required to accurately predict the behaviour of TS. However, the multiconfigurational CAS methods are computationally demanding and cannot be routinely employed for the calculations. A previous benchmarking study has demonstrated that the single reference DFT-based PBE method is still better to predict the geometry of TS using nudged elastic band (NEB) calculations and multireference methods determines the activation energy values close to the experiments [42].

In the present investigation, the geometry of all the TS structures was obtained with the climbing image NEB (CI-NEB) calculations implemented in the Quantum Espresso package analogous to the earlier reports [33,36,42,45]. Thereafter, the single point energy calculations were performed at the (8,8)-CASPT2/6-311++G** level for the singlet biradicaloid TS and the photoproduct. The TBR barrier was accounted by computing the electronic energy difference between for the TS and the corresponding photoproduct.

The TBR barrier can be calculated using the following equation:


[3] $$TBR_{\text{CI-NEB}}=E_{\text{TS(CI-NEB)}}^{\text{CASPT2}}-E_{\text{photoproduct}}^{\text{CASPT2}},$$

where TBR_Cl-NEB_ designates the TBR barrier calculated by using the TS geometry obtained with CI-NEB, 
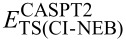
 is the electronic energy computed with the (8,8)-CASPT2/6-311++G** level for the TS structure obtained with CI-NEB and 
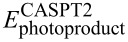
 is the electronic energy of photoproduct calculated with (8,8)-CASPT2/6-311++G**.

The geometries of TS obtained with CI-NEB method were re-optimized at the PBE/6-311++G** level employing Gaussian 16 [44]. The nature of the point structure was verified as a saddle point with the harmonic frequency calculations at the same level of theory. Afterwards, the TBR barrier was also predicted by performing a single point energy calculation at (8,8)-CASPT2/6-311++G** utilizing the TS geometries obtained with PBE/6-311++G**. The energy barrier for back isomerization reaction was calculated as:


[4] $$TBR_{\text{Gaussian16}}=E_{\text{TS(Gaussian16)}}^{\text{CASPT2}}-E_{\text{photoproduct}}^{\text{CASPT2}},$$

where *TBR*_Gaussian16_ designates the TBR barrier calculated by using the TS geometry obtained with Gaussian16, and 
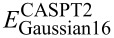
 is the electronic energy of TS computed with the (8,8)-CASPT2/6-311++G** for TS structure obtained with Gaussian16. The TBR barriers accounted by using the TS geometries obtained with PBE functional using CI-NEB method are systematically compared with those obtained with PBE/6-311++G** level using Gaussian16.

The vibrational corrections obtained in the TS with the single-reference DFT functionals are inappropriate to include the electronic energies computed with (8,8)-CASPT2/6-311++G** due to cusp in energy landscape obtained with the single-reference methods [42]. Further, the vibrational analyses are computationally expensive with multireference methods and even inaccurate due to numerical differentiation [42]. A previous benchmarking study has also suggested that the electronic energy difference (Δ*E^‡^*) accounts the TBR barrier closer to the experimental enthalpy difference (Δ*H*^‡^) [42]. Therefore, the TBR barriers were predicted with the difference in electronic energy of the TS and photoisomer (Δ*E^‡^*) akin to the earlier study. All the single point electronic energy calculations at the DLPNO-CCSD(T)/Def2TZVP and (8,8)-CASPT2/6-311++G** level were carried out using ORCA [46].

Intrinsic reaction coordinate (IRC) calculations have also been performed to validate the interconnection of the TS with the expected local minima on the PES. To confirm the reversible photoswitching in the studied BBD-based switches, ab initio molecular dynamics (AIMD) simulations were carried out. The simulations were initiated using the singlet biradicaloid TS of the IIa photoswitch. The simulations were performed for 2 ps with the time step of 1 fs by employing the CP2K package [47]. The Nosé–Hoover thermostat was employed to regulate the temperature of the systems at 300 K during the entire simulations. The AIMD simulations were executed employing the DZVP basis set, Goedecker–Teter–Hutter (GTH) pseudopotential and Perdew–Burke–Ernzerhof (PBE) functional along with the inclusion of the Grimme’s D3 dispersion correction.

## Supporting Information

File 1 Additional tables, figures and Cartesian coordinates.

File 2 AIMD simulation movie.

## Data Availability

All data that supports the findings of this study is available in the published article and/or the supporting information to this article.
