# Supplementary material for: Novel route to enhance the thermo-optical performance of bicyclic diene photoswitches for solar thermal batteries
Source: Beilstein J Org Chem. 2024 May 13;20:1053–68. doi: 10.3762/bjoc.20.93 (PMC11106670; doi:10.3762/bjoc.20.93)
Supplement: File 1 — Additional tables, figures and Cartesian coordinates. [file Beilstein_J_Org_Chem-20-1053-s001.pdf]

BEILSTEIN JOURNAL OF ORGANIC CHEMISTRY

Supporting Information

for

Novel route to enhance the thermo-optical performance of bicyclic diene photoswitches for solar thermal batteries

Akanksha Ashok Sangolkar, Rama Krishna Kadiyam and Ravinder Pawar

Beilstein J. Org. Chem. **2024,** *20,* 1053–1068. doi:10.3762/bjoc.20.93

Additional tables, figures and Cartesian coordinates

License and Terms: This is a supporting information file under the terms of the Creative Commons Attribution License (https://creativecommons.org/licenses/by/4.0). Please note that the reuse, redistribution and reproduction in particular requires that the author(s) and source are credited and that individual graphics may be subject to special legal provisions.
The license is subject to the *Beilstein Journal of Organic Chemistry* terms and conditions: (https://www.beilstein-journals.org/bjoc/terms)

S1. Optimized geometries of the transition states along the displacement vectors.

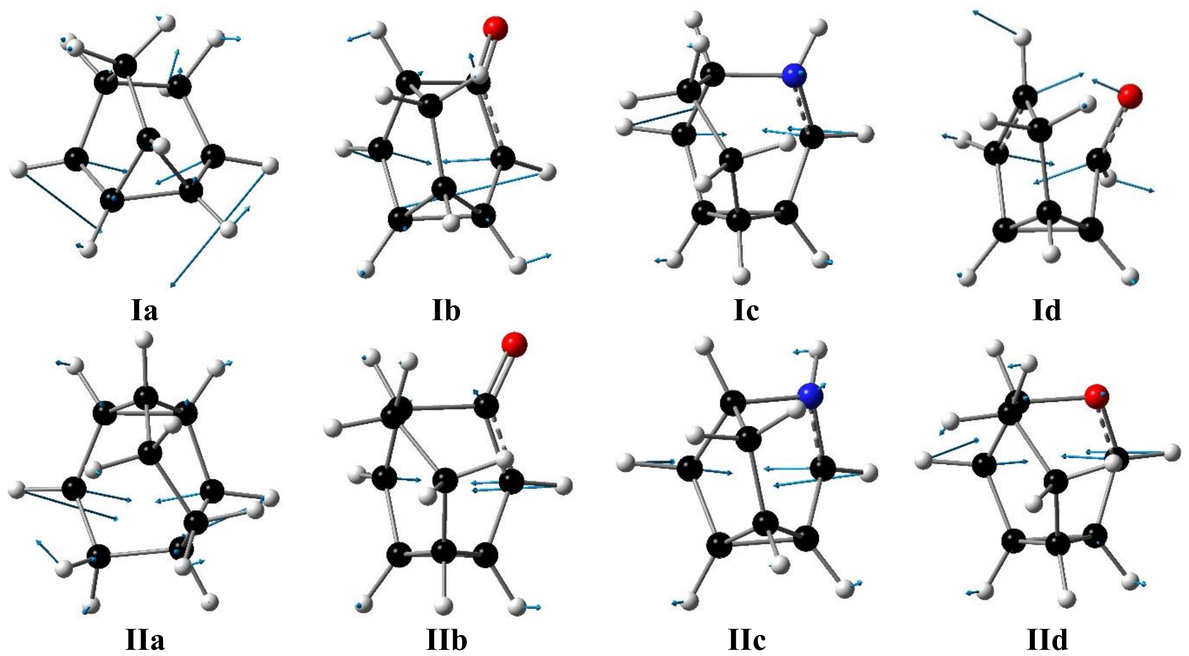

Figure S1: Optimized geometries of TS involved in the back isomerization of the photoproducts to the parent bicyclic dienes illustrating the displacement vectors at the PBE/6-311++G** level of theory (TS for type Id photoswitch is associated with the thermal degradation to carbonyl compound).

S2. Solar energy conversion efficiencies and the reality check for the energy storage efficiencies of the studied BBD-based photoswitches in gas phase.

Table S1: The calculated energy of the photon involved for the first important electronic excitation (with oscillatory strength higher than 0.01), sum of storage energy (SE) and the TBR barrier, and the solar energy conversion efficiency of the studied BBD-based photoswitches.

System	E_{photon} (kJ/mol)	SE + TBR barrier (kJ/mol)	$\eta_{efficiency}$
**Ia**	562.15	290.79	10.06
**Ib**	483.97	248.29	7.78
**Ic**	479.18	313.57	10.08
**IIa**	579.08	293.49	9.36
**IIb**	507.19	280.26	7.99
**IIc**	475.43	305.76	10.31
**IId**	497.66	282.86	8.89

S3. Effect of solvation on the thermochemical properties

Table S2: Effect of solvation on the thermochemical properties of the studied BBD-based photoswitches. The energy storage capacities were calculated at DLPNO-CCSD(T)/Def2TZVP level for the geometries optimized with the M062X/6-311++G**. The TBR barrier were calculated at (8,8)-CASPT2/6-311++G** level for the geometries optimized with the PBE/6-311++G** (SMD solvation model).

Medium	Ia	Ic	IIa	IIc
	**Storage energy (kJ/mol)**			
**Gas**	141.15	158.38	156.35	165.74
**Cyclohexane**	143.57	158.61	158.95	167.31
**Toluene**	143.96	158.60	159.35	167.49
**Dichloromethane**	145.72	158.56	161.05	168.32
**Ethanol**	146.14	158.81	161.46	168.88
**Acetonitrile**	146.22	158.57	161.55	168.72
	**ESD (MJ/kg)**			
**Gas**	1.33	1.48	1.30	1.37
**Cyclohexane**	1.35	1.48	1.32	1.38
**Toluene**	1.36	1.48	1.33	1.38
**Dichloromethane**	1.37	1.48	1.34	1.39
**Ethanol**	1.38	1.48	1.34	1.39
**Acetonitrile**	1.38	1.48	1.35	1.39
	**TBR barrier (kJ/mol)**			
**Gas**	149.64	155.19	137.14	140.02
**Cyclohexane**	148.27	160.99	137.44	139.83
**Toluene**	148.17	153.44	137.23	139.83
**Dichloromethane**	145.26	162.67	137.05	139.89
**Ethanol**	145.93	139.14	135.82	139.81
**Acetonitrile**	146.12	136.36	137.50	139.70

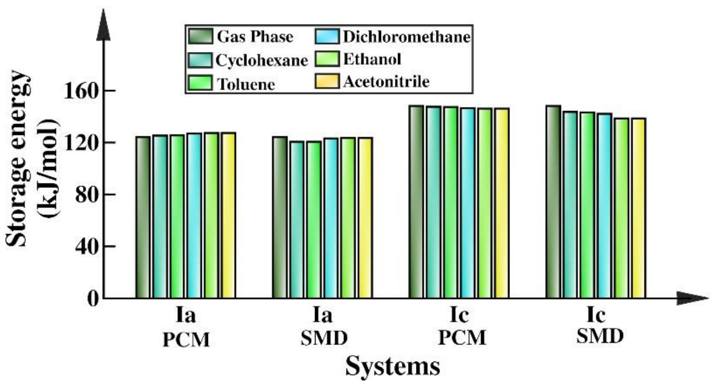

Figure S2: Effect of solvation model on storage energies of type Ia and Ic photoswitches in different polarity solvents calculated at the M062X/6-311++G** level of theory

S4. Effect of solvation on the photophysical properties of the studied BBD-based photoswitches

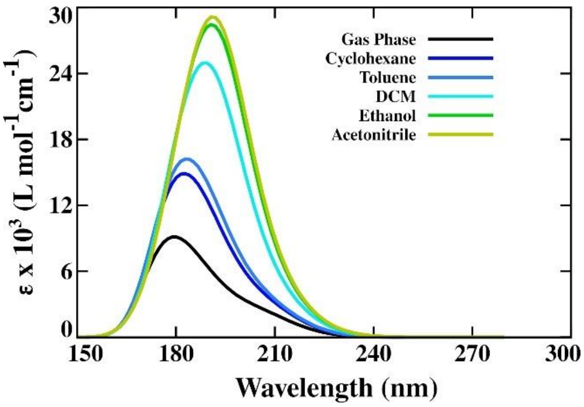

(a) IIa

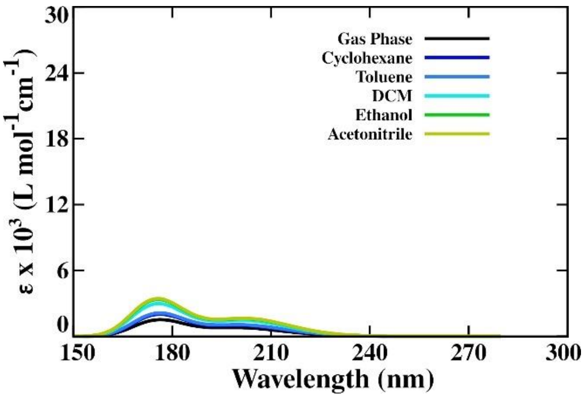

(b) IIa

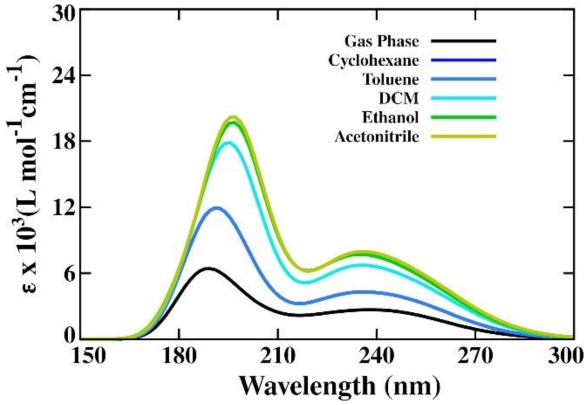

(c) IIc

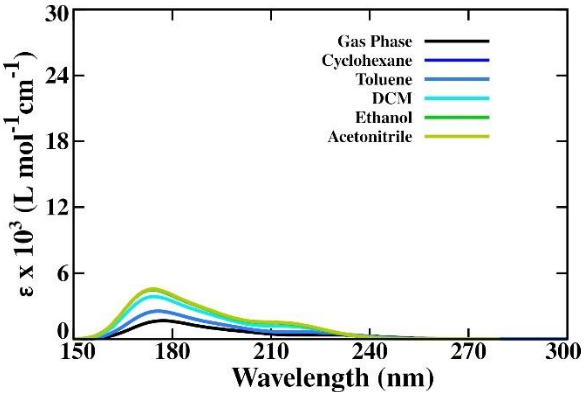

(d) IIc

Figure S3: Effect of different polarity solvents on the optical absorption spectra of dienes and photoproducts of IIa and IIc photoswitches (M062X/6-311++G**).

Table S3: Spectral data for the optical absorption of the diene and photoproducts of Ia and Ic photoswitches in the presence of different polarity solvents (M062X/6-311++G** considering SMD solvation model).

Systems	Medium	λ_{onset} (nm)	*f*	λ (nm)	ε ($Lmol^{-1}cm^{-1}$)	λ_{onset} (nm)	*f*	λ (nm)	ε ($Lmol^{-1}cm^{-1}$)
				**Diene**				**Photoproduct**	
**Ia**	**Gas**	241.12	0.029	212.80	2151.00	216.48	0.010	185.09	1377.58
	**Cyclohexane**	244.72	0.022	217.36	2481.97	218.40	0.013	184.90	1812.15
	**Toluene**	245.44	0.026	217.68	2631.70	218.72	0.010	193.87	1198.71
	**Dichloromethane**	250.40	0.044	219.75	3758.86	220.00	0.015	194.01	1579.31
	**Ethanol**	252.00	0.050	220.51	4228.76	220.64	0.017	194.07	1751.84
	**Acetonitrile**	252.00	0.051	220.64	4380.79	220.96	0.018	194.17	1790.76
**Ic**	**Gas**	295.60	0.043	249.65	2375.97	251.20	0.011	177.38	1669.30
	**Cyclohexane**	299.00	0.061	250.45	3608.89	247.60	0.014	224.13	609.59
	**Toluene**	299.50	0.061	250.52	3620.59	247.60	0.014	224.08	609.70
	**Dichloromethane**	303.50	0.023	262.01	3659.86	244.00	0.024	218.04	1105.17
	**Ethanol**	303.50	0.034	260.50	4404.56	242.20	0.030	215.68	1398.38
	**Acetonitrile**	305.00	0.034	261.16	4784.67	242.92	0.031	216.08	1425.70

Table S4: Spectral data for the optical absorption of the diene and photoproducts of IIa and IIc photoswitches in the presence of different polarity solvents (M062X/6-311++G** considering SMD solvation model).

Systems	Medium	λ_{onset} (nm)	f	λ (nm)	ε ($Lmol^{-1}cm^{-1}$)	λ_{onset} (nm)	f	λ (nm)	ε ($Lmol^{-1}cm^{-1}$)
		Diene				Photoproduct			
IIa	Gas	233.92	0.022	206.58	2697.24	229.96	0.010	172.85	1376.49
	Cyclohexane	236.08	0.031	208.27	3564.67	232.12	0.013	204.69	951.39
	Toluene	236.80	0.034	208.56	3833.06	232.48	0.013	204.81	1008.82
	Dichloromethane	240.04	0.057	210.62	6334.85	234.28	0.020	205.20	1383.39
	Ethanol	241.12	0.062	211.16	7580.02	235.00	0.023	205.36	1546.73
	Acetonitrile	241.48	0.063	211.30	7982.72	235.00	0.024	205.39	1578.63
IIc	Gas	292.20	0.029	251.62	2162.12	247.40	0.013	202.79	1292.60
	Cyclohexane	296.20	0.042	252.51	2994.26	247.60	0.016	200.60	1758.85
	Toluene	297.40	0.045	252.57	3211.26	247.60	0.016	200.05	1877.45
	Dichloromethane	302.50	0.066	252.98	4787.90	246.00	0.014	222.39	913.87
	Ethanol	303.00	0.014	263.95	3286.29	245.20	0.019	220.07	1273.94
	Acetonitrile	304.50	0.014	264.66	3444.76	245.60	0.020	220.24	1283.45

CARTESIAN COORDINATES

Ia-R1

C	-1.65384700	0.30952200	-0.36945100
C	-0.86544900	1.35879000	-0.13630600
C	0.55044300	1.13653100	0.37408700
H	0.97655600	2.04517700	0.79899500
C	1.36368600	0.53603700	-0.77272200
C	1.19356200	-0.78466400	-0.78795500
C	0.26307400	-1.19946400	0.34095100
H	0.47589600	-2.19208500	0.74137300
C	0.51102700	-0.05891200	1.34469000
H	1.89579400	1.12486900	-1.50960800
H	1.56900000	-1.46648400	-1.54159300
H	1.47713500	-0.18845000	1.83629600
H	-0.27525200	0.03593200	2.09611700
H	-1.21216600	2.36828000	-0.33139800
H	-2.65939100	0.45705300	-0.75210100
C	-1.20441100	-1.11625000	-0.13954100
H	-1.33464800	-1.69103800	-1.06446200
H	-1.86143100	-1.58279700	0.60386800

Ia-TS

C	0.64726900	1.45624500	-0.10263600
C	-0.81347000	1.15146300	-0.21558100
C	-1.18890800	-0.28735700	-0.76710100
H	-2.05519000	-0.35711500	-1.43394800
C	-1.29023100	0.13880600	0.72508300
C	-0.06771200	-0.46287800	1.37215800
C	0.88725700	-0.89689900	0.28701300
H	1.55158500	-1.72374900	0.57525200
C	-0.04832600	-1.26658500	-0.91261200
H	-2.24740500	0.22600600	1.24469100
H	-0.16457200	-1.05751000	2.28636100
H	-0.42145200	-2.29582500	-0.78910200
H	0.45717700	-1.20350400	-1.89140300
H	-1.48004600	1.95526000	-0.53773900
H	0.95702200	2.42272100	-0.52426900
C	1.66616600	0.36740000	-0.12579200
H	2.45714100	0.53860100	0.61958100
H	2.19346500	0.29394800	-1.11260700

Ia-P1

C	-0.53969300	-1.20081000	-0.27611800
C	0.88156100	-0.83572200	-0.74300900
C	1.23518100	0.60099400	-0.42303900
H	2.17410200	0.94163200	-0.84271400
C	1.28845800	-0.42165500	0.66665200
C	-0.19618800	-0.51626800	1.07260100
C	-0.96097300	0.74578500	0.50384400
H	-1.61290100	1.30409500	1.17434400
C	0.12366800	1.59879100	-0.15916700
H	2.16401900	-0.67820900	1.24605500
H	-0.49576500	-0.93063100	2.03075100
H	0.49097300	2.37544900	0.51855600
H	-0.24906200	2.08417000	-1.06511200
H	1.45852600	-1.38726200	-1.47387700
H	-0.75566600	-2.26705300	-0.33022400
C	-1.63780200	-0.16334100	-0.55425200
H	-2.61033300	-0.52736000	-0.21627700
H	-1.72916500	0.23853400	-1.56658100

Ia-P2

C	1.64982300	-0.32722400	0.48076900
C	0.63039000	-1.06482000	-0.31162000
C	-0.80886900	-1.15328900	0.12588400
H	-1.28956000	-2.12192300	0.09426900
C	-0.44885700	-0.42514100	-1.16076400
C	-0.79224100	1.00352600	-0.90458200
C	-1.25350300	1.18908800	0.33050900
H	-1.51394100	2.14814900	0.76147400
C	-1.32102600	-0.10397300	1.11011600
H	-0.64398900	-0.85901200	-2.13343000
H	-0.65152900	1.78469800	-1.64177200
H	-2.34091900	-0.32535900	1.43738000
H	-0.69908200	-0.05227100	2.01209100
H	1.00469700	-1.99882600	-0.72246900
H	1.94579400	-0.80643900	1.41306400
C	2.22198300	0.81841500	0.13165700
H	1.94726300	1.32511300	-0.78714700
H	2.97506600	1.28637200	0.75471700

Ib-R1

C	-0.83172800	1.34779900	-0.28197800
C	0.47054300	1.63343500	-0.17476200
C	1.45256600	0.56189000	0.25971500
H	2.40039300	0.99489300	0.57777300
C	1.58664200	-0.47191700	-0.86574500
C	0.60851400	-1.36530000	-0.76140300
C	-0.26750000	-1.03603600	0.44518200
H	-0.75948000	-1.89435400	0.90053600
C	0.74948800	-0.29380600	1.33453800
H	2.32497700	-0.40858500	-1.65423600
H	0.39110100	-2.17079900	-1.45003200
H	1.44445700	-1.00697700	1.78090000
H	0.28787300	0.31021000	2.11707700
H	0.83939700	2.62659400	-0.41288900
H	-1.57013600	2.07375800	-0.60238100
C	-1.33278200	-0.02888400	0.00214400
O	-2.49663000	-0.32597800	-0.12536200

Ib-TS

C	-0.52377500	-1.25650600	-0.33774200
C	0.98758400	-1.13320500	-0.47256000
C	1.59595500	0.27108200	-0.59100800
H	2.56584600	0.33872300	-1.08718400
C	1.48870500	-0.44287500	0.75368000
C	0.16834300	0.06685100	1.31301600
C	-0.43749400	1.04394500	0.39160000
H	-1.06370400	1.84086400	0.80425700
C	0.63627300	1.44310900	-0.63306300
H	2.32608500	-0.74977600	1.38234100
H	-0.20534200	-0.13972100	2.31662100
H	1.15070500	2.36638200	-0.31808800
H	0.20579900	1.61927600	-1.63132400
H	1.53270800	-1.96259800	-0.92473600
H	-0.99143300	-2.21051600	-0.61163600
C	-1.36897800	-0.12104400	-0.16644000
O	-2.60004100	-0.04134700	-0.18439400

Ib-P1

C	-0.34223600	-1.20636700	-0.05625500
C	0.95525600	-0.88121900	-0.83722800
C	1.39550300	0.55574500	-0.65946800
H	2.22123800	0.85265500	-1.29448400
C	1.67212500	-0.43328400	0.42503600
C	0.31469400	-0.46851700	1.15881600
C	-0.51962300	0.80754400	0.75173100
H	-1.01134000	1.40868300	1.51424800
C	0.38769200	1.59573100	-0.21170100

Ib-P2

C	1.05844100	1.05904500	-0.53794600
C	-0.14834300	1.33928600	0.29203400
C	-1.51256500	0.81435600	-0.08510700
H	-2.34609900	1.50116700	-0.02342100
C	-0.82824700	0.30873100	1.17154500
C	-0.52992500	-1.12881300	0.90405600
C	-0.91596200	-1.49012400	-0.31874500
H	-0.75144900	-2.46523500	-0.76083800
C	-1.57978700	-0.35404200	-1.06297400

H	2.64678500	-0.69687100	0.80883000
H	0.21960800	-0.84832200	2.17041600
H	0.89801000	2.40727600	0.31402300
H	-0.18390000	2.02824200	-1.03606300
H	1.32918000	-1.47649600	-1.65856500
H	-0.61929700	-2.25842600	-0.04089300
C	-1.40155300	-0.12273300	-0.08325800
O	-2.53393000	-0.06226800	-0.46294400

Ic_R$_1$

C	-1.62877900	0.15117700	-0.34387200
C	-0.94317800	1.28487500	-0.13012100
C	0.49073800	1.16324000	0.36264400
H	0.87887900	2.09871800	0.76291900
C	1.27429900	0.59367800	-0.81317300
C	1.18343200	-0.73923100	-0.81268700
C	0.30609400	-1.17030400	0.35202000
H	0.50851800	-2.16204600	0.75540000
C	0.54193300	-0.02250500	1.34364500
H	1.70752800	1.20259800	-1.59785200
H	1.52531000	-1.40472200	-1.59614900
H	1.53072200	-0.11980900	1.79311400
H	-0.22388300	0.02642000	2.11793700
H	-1.39300100	2.24581500	-0.33760300
H	-2.63591100	0.15195300	-0.74421500
N	-1.11436500	-1.10609600	-0.04021700
H	-1.44484700	-1.86183900	-0.62276900

Ic-P$_1$

C	-0.54660200	-1.16248100	-0.28332200
C	0.88335000	-0.82574100	-0.73433900
C	1.23123800	0.60710700	-0.39879400
H	2.17375900	0.95282800	-0.80515200
C	1.27046900	-0.42095000	0.68715900
C	-0.21784800	-0.51610600	1.07628300
C	-0.98140700	0.71496300	0.44872300
H	-1.72079500	1.24715500	1.04616300
C	0.10317600	1.59531900	-0.16031300
H	2.13570600	-0.68779600	1.27598600
H	-0.54104600	-0.92679200	2.02863300
H	0.43436400	2.36690700	0.54157500
H	-0.26403500	2.06756500	-1.07236800
H	1.46068900	-1.37607400	-1.46507200
H	-0.82848500	-2.20867200	-0.39621200
N	-1.54808100	-0.13183300	-0.65540500
H	-2.46784800	-0.46496000	-0.37810400

Id-R$_1$

C	-1.60763500	0.05407000	-0.37589100
C	-0.99334500	1.21980600	-0.15349200
C	0.42866400	1.17574400	0.37862400
H	0.75611600	2.12640900	0.79559100
C	1.28321900	0.64064800	-0.76631800
C	1.22233300	-0.69204000	-0.78643000
C	0.31496300	-1.14851200	0.33899200
H	0.48815300	-2.15265400	0.71861000
C	0.47934400	-0.01599500	1.35348200
H	1.75046700	1.27112000	-1.51335600
H	1.61774400	-1.34737700	-1.55096000
H	1.45364600	-0.09613000	1.83628700
H	-0.31375400	0.00324100	2.10102900
H	-1.50042400	2.14806200	-0.37771600
H	-2.61032100	-0.03694800	-0.77392000
O	-1.05086000	-1.16475700	-0.14617000

Id-P$_1$

C	-0.47331400	-1.17745200	-0.30505700
C	0.93873900	-0.74941200	-0.73569100
C	1.17341900	0.69942800	-0.36897200
H	2.09167700	1.12471500	-0.75416700
C	1.27137500	-0.34147200	0.69966800
C	-0.20941900	-0.55296500	1.06950900

H	-1.13290900	0.62397600	2.16109500
H	-0.03133000	-1.77137700	1.61907400
H	-2.61384300	-0.59644400	-1.32396400
H	-1.05175700	-0.13353100	-1.99782900
H	-0.16730800	2.33967700	0.71211100
H	1.25327400	1.62773600	-1.43908800
C	1.94969800	0.14641200	-0.22944900
O	2.73519500	-0.66188500	0.03154600

Ic-TS

C	-0.77742700	-1.30832600	-0.17010400
C	0.70058900	-1.14126200	-0.40465500
C	1.22749500	0.30686700	-0.67629700
H	2.10688800	0.37319500	-1.32401000
C	1.24822900	-0.31663500	0.70760800
C	-0.01537600	0.16900800	1.42918400
C	-0.85843500	0.87211000	0.32309100
H	-1.56960200	1.63585000	0.66520700
C	0.19048300	1.40874300	-0.70268000
H	2.16150400	-0.59432400	1.23676000
H	0.17833900	0.79190700	2.31855000
H	0.62921800	2.35119200	-0.33900600
H	-0.25200900	1.58562400	-1.69541500
H	1.24653500	-1.97240400	-0.85834100
H	-1.23079800	-2.29211000	-0.34439800
N	-1.57255000	-0.21367500	-0.36671800
H	-2.55557200	-0.32623800	-0.12919800

Ic-P$_2$

C	-1.55315600	0.43771900	0.09221300
C	-0.76458600	-0.80332600	-0.02737900
C	0.53296800	-0.87437700	-0.80414700
H	0.65716900	-1.73663100	-1.44477700
C	0.56057200	-1.01655200	0.69686000
C	1.26530700	0.20198900	1.19221200
C	1.63495000	0.99841100	0.18955000
H	2.12207500	1.95935200	0.30263600
C	1.26145500	0.41974300	-1.15773400
H	0.70380300	-1.96895000	1.19025200
H	1.42483000	0.40382500	2.24408200
H	2.15174200	0.21476900	-1.75976100
H	0.63767500	1.10608600	-1.74051700
H	-1.39041500	-1.68381700	-0.11096700
H	-0.99824600	1.36197300	0.28002100
N	-2.81780600	0.40326300	-0.01992300
H	-3.20904700	1.33891100	0.08903600

Id-TS

C	-0.92467000	-0.91409800	-0.15081200
C	0.50732200	-1.05633400	-0.69294200
C	1.28342800	0.23638500	-0.61031600
H	2.21925800	0.29555900	-1.16686500
C	1.19903300	-0.66507000	0.61103400
C	-0.06349100	-0.16730100	1.30256500
C	-0.55375800	0.99354900	0.60634700
H	-1.41129600	1.57941600	0.93471900
C	0.45897500	1.49278600	-0.36989500
H	2.04858200	-1.14247200	1.09781300
H	-0.42948700	-0.47715700	2.28021600
H	1.07135700	2.26320600	0.14417600
H	0.00787600	1.94172700	-1.26422200
H	0.78258100	-1.83168100	-1.41324800
H	-1.31375700	-1.91300200	0.15884900
O	-1.80201800	-0.02938600	-0.61841600

Id-P$_2$

C	-1.56265000	0.48588800	0.08286800
C	-0.80334500	-0.78021500	-0.01816700
C	0.50153500	-0.88933800	-0.78799200
H	0.59657200	-1.76347100	-1.41712100
C	0.52061200	-1.00913000	0.70902500
C	1.24866300	0.20267400	1.19189200

C	-1.04909500	0.60984500	0.43637700
H	-1.87891100	1.04627800	0.99031200
C	-0.03808400	1.58854600	-0.14060600
H	2.14422200	-0.55874200	1.29706800
H	-0.52008600	-1.00538700	2.00495400
H	0.20749000	2.37786500	0.57614500
H	-0.43123300	2.03958800	-1.05314000
H	1.56465100	-1.24709100	-1.46388400
H	-0.73687800	-2.22101100	-0.46863000
O	-1.51533300	-0.25191500	-0.63250300

IIa-R$_1$

C	1.35589900	1.24658900	-0.08575900
C	0.11128000	1.71622300	-0.01736500
C	-1.13461400	0.86578600	0.15866500
H	-1.99126600	1.53037400	0.28310100
C	-1.01363900	-0.04772300	1.36013100
C	-0.17407000	-1.07506200	1.27451600
C	0.64001000	-1.22769900	0.01205700
H	1.10217600	-2.21776000	-0.00836200
C	-0.25754300	-1.08136900	-1.24207600
C	-1.36829900	-0.01955400	-1.08583300
H	-1.60652300	0.14190500	2.24838000
H	-0.03909800	-1.77314900	2.09402800
H	-0.71440000	-2.04888800	-1.46006700
H	0.38224300	-0.83838000	-2.09492500
H	-2.33940300	-0.50767300	-0.97178300
H	-1.42257400	0.61032200	-1.97689800
H	-0.03678700	2.78934900	-0.09586500
H	2.16440300	1.96407000	-0.20235000
C	1.78371300	-0.19591800	-0.01018100
H	2.41971700	-0.32919500	0.87445600
H	2.42508800	-0.40861200	-0.87463900

IIa-P$_1$

C	-1.24326800	0.82093200	-0.41256000
C	-1.36456300	-0.70597900	-0.55902600
C	-0.12215700	-1.49494000	-0.23344100
H	-0.31885700	-2.55571200	-0.36062100
C	-0.91269500	-0.90710500	0.89410200
C	-0.65037800	0.60998900	0.99795700
C	0.72654400	1.13541600	0.47091100
H	1.10415500	1.98684800	1.04014500
C	1.78973000	0.05250800	0.36340700
C	1.31883300	-1.10177800	-0.52566700
H	-1.36845200	-1.51292700	1.66720900
H	-1.07124400	1.11205300	1.86681400
H	1.99680100	-0.32935500	1.36985500
H	2.72417100	0.46931600	-0.02438400
H	1.95770800	-1.97238900	-0.35386800
H	1.42938800	-0.84265200	-1.58276900
H	-2.18139100	-1.19626800	-1.07458900
H	-2.19648700	1.33372000	-0.53769700
C	0.04400600	1.54053200	-0.87358700
H	-0.10441100	2.61969200	-0.94700400
H	0.51231300	1.19022200	-1.79566000

IIb-R$_1$

C	0.90104300	1.44906200	-0.04935800
C	-0.40637700	1.71849200	0.01819400
C	-1.50833600	0.69461900	0.16726700
H	-2.45565600	1.22113100	0.28731600
C	-1.27692600	-0.23033400	1.34489100
C	-0.29093200	-1.11541400	1.25482000
C	0.55912600	-1.11611600	0.00608600
H	1.20755200	-1.99106600	-0.02813400
C	-0.33635800	-1.07305200	-1.26685100
C	-1.58641700	-0.18708500	-1.10957200
H	-1.92433600	-0.16797600	2.21201400
H	-0.06819600	-1.81741500	2.04966100
H	-0.64049800	-2.09708000	-1.48862800
H	0.27292500	-0.73762100	-2.10899900
H	-2.48013000	-0.80755400	-1.02029000

C	1.64713100	0.96996100	0.17827700
H	2.15733200	1.92012700	0.27732500
C	1.26731300	0.37798200	-1.16097400
H	0.63413000	-1.95646900	1.21934400
H	1.40242700	0.41802100	2.24164300
H	2.15633700	0.13089400	-1.74888300
H	0.67335600	1.07138200	-1.76486300
H	-1.45016400	-1.64591200	-0.09492700
H	-0.97951800	1.40693100	0.25797600
O	-2.76325400	0.53394700	-0.01750800

IIa-TS

C	-1.00851600	1.29174000	-0.65538900
C	-1.60285000	-0.04506200	-0.29854700
C	-0.70931000	-1.33172300	-0.31763700
H	-1.27341100	-2.25575500	-0.49400300
C	-1.09069400	-0.53559500	0.97584400
C	-0.03869200	0.40888000	1.47666000
C	1.07548700	0.75131400	0.51924100
H	1.84402700	1.35046700	1.03284800
C	1.72215700	-0.57567200	0.03523900
C	0.72214700	-1.36804700	-0.83235500
H	-1.75458700	-1.01788500	1.69743600
H	0.25385100	0.30264000	2.52994800
H	2.00688300	-1.17494300	0.91716800
H	2.64658300	-0.38171000	-0.53451700
H	1.04601100	-2.41936100	-0.90331800
H	0.74744700	-0.97595100	-1.86539200
H	-2.65246700	-0.20078400	-0.55979600
H	-1.58275100	1.88089800	-1.38369900
C	0.45618700	1.55339900	-0.63187300
H	0.62601500	2.62573100	-0.43608300
H	0.93689900	1.37124900	-1.62769400

IIa-P$_2$

C	-1.64548800	-0.21514800	-0.75004500
C	-0.91305500	-1.11585100	0.18227800
C	0.55658600	-1.40747400	0.09330900
H	0.83507000	-2.43448000	0.30300000
C	0.03046000	-0.61913400	1.27382700
C	0.43156800	0.80442200	1.36282700
C	1.00787400	1.46149600	0.35575400
H	1.27315200	2.50641100	0.48008900
C	1.27742100	0.81753200	-0.97359700
C	1.49877300	-0.70012000	-0.86070700
H	-0.05160100	-1.13784600	2.22115700
H	0.24351100	1.31389100	2.30283300
H	2.15324200	1.27423300	-1.44181000
H	0.43343100	1.02121600	-1.64162800
H	2.51620300	-0.87424200	-0.49622300
H	1.43771900	-1.15410100	-1.85397200
H	-1.50515600	-1.97542900	0.48548800
H	-1.63840700	-0.50443500	-1.80028200
C	-2.32479300	0.86158900	-0.37298000
H	-2.33979700	1.17678700	0.66487000
H	-2.87344600	1.46412400	-1.08752100

IIb-TS

C	-0.62613100	-1.33125200	-0.51656200
C	0.90496000	-1.38111200	-0.47458600
C	1.71431000	-0.09934200	-0.47438900
H	2.76208300	-0.25666300	-0.74489400
C	1.33344900	-0.79230600	0.83838600
C	-0.00690200	-0.38412900	1.39256700
C	-0.68925000	0.76929100	0.80024600
H	-1.47595300	1.18893000	1.43899600
C	0.20066800	1.84264600	0.16888000
C	1.12447500	1.24090300	-0.89922000
H	2.08780200	-1.22840400	1.49620800
H	-0.40165300	-0.88029400	2.28221800
H	0.79870500	2.32412200	0.96270500
H	-0.43668800	2.62430800	-0.27334700
H	1.94152800	1.94284100	-1.12689600

H	-1.71709900	0.45135400	-1.98556200
H	-0.71570900	2.75811700	-0.05201100
H	1.62690700	2.24929400	-0.14758100
C	1.49640200	0.08798000	0.00544100
O	2.69836100	-0.05376200	0.00708800

IIb-P1

C	-0.29549100	-1.43368200	-0.08160500
C	1.06439200	-1.12475900	-0.74946500
C	1.50784400	0.31240300	-0.66574600
H	2.46593100	0.42940800	-1.16172900
C	1.60969000	-0.54708600	0.55690900
C	0.21799500	-0.72389900	1.20240600
C	-0.80904700	0.43505700	0.99020100
H	-1.51400800	0.57001600	1.81263700
C	-0.19151900	1.75290600	0.53492600
C	0.61009300	1.54034100	-0.75149400
H	2.54918900	-0.75872800	1.04893600
H	0.18486600	-1.23501900	2.16003700
H	0.46826100	2.12149700	1.32687600
H	-0.97441600	2.49717500	0.37596300
H	1.23089200	2.41723700	-0.94627400
H	-0.07335200	1.44254300	-1.60103800
H	1.54415600	-1.80359500	-1.44097000
H	-0.58117500	-2.48378200	-0.09928600
C	-1.40253800	-0.38339600	-0.17605900
O	-2.39610700	-0.27050800	-0.82944900

IIc-R1

C	1.44560000	1.06735200	-0.21467200
C	0.25513000	1.67804100	-0.13832300
C	-1.05259000	0.95209200	0.13428300
H	-1.84296800	1.69422900	0.25624200
C	-0.89729900	0.14546100	1.40623000
C	-0.12738000	-0.94118500	1.36065300
C	0.56436700	-1.23953300	0.05035800
H	1.01670200	-2.23217100	0.06726800
C	-0.42741700	-1.17206300	-1.12608800
C	-1.43323800	-0.00252200	-1.01164500
H	-1.36845500	0.47790300	2.32566400
H	0.06838300	-1.55191900	2.23567500
H	-0.96978300	-2.11925000	-1.16387600
H	0.15861800	-1.09597300	-2.04434900
H	-2.43747100	-0.39031400	-0.81936400
H	-1.47175000	0.55759900	-1.94863300
H	0.23692200	2.75542100	-0.24307700
H	2.34774400	1.65762000	-0.33924200
N	1.67914500	-0.30252100	-0.19295700
H	2.54500700	-0.56135400	0.25961300

IIc-P1

C	-1.11961000	0.92245300	-0.42727600
C	-1.42012900	-0.57933500	-0.55581700
C	-0.25571500	-1.47013700	-0.20650500
H	-0.54367500	-2.51073900	-0.31970400
C	-0.99651600	-0.79883400	0.90729600
C	-0.58996400	0.68516600	0.99310800
C	0.80647800	1.07593300	0.41615200
H	1.24151400	1.95363600	0.89887300
C	1.79054000	-0.07741400	0.34919000
C	1.21327000	-1.20495400	-0.51065400
H	-1.51574500	-1.34281800	1.68472400
H	-0.95552000	1.25015700	1.84690700
H	1.97037500	-0.43453300	1.36956200
H	2.74736200	0.25600500	-0.06052400
H	1.77601200	-2.12402000	-0.32881300
H	1.32654800	-0.95106700	-1.56562900
H	-2.27672700	-0.99158600	-1.07355500
H	-1.97137500	1.57183800	-0.62668800
N	0.20396700	1.37633700	-0.92817000
H	0.20334500	2.37149600	-1.12092500

IId-R1

H	0.55932100	1.10444400	-1.83739100
H	1.36122800	-2.29839300	-0.84964400
H	-1.12786000	-2.16899900	-1.01709900
C	-1.44028300	-0.19134200	-0.24654400
O	-2.64503600	-0.04900400	-0.48294000

IIb-P2

C	1.48935900	0.96277600	-0.55400400
C	0.47450000	1.46085200	0.41540600
C	-1.01498700	1.30559100	0.15410200
H	-1.62215500	2.15198800	0.45177500
C	-0.36213300	0.52967300	1.26580000
C	-0.30461400	-0.94917700	1.11539400
C	-1.08914400	-1.59293400	0.25251300
H	-1.04650100	-2.67598400	0.19391500
C	-2.05110300	-0.86553100	-0.65356400
C	-1.51116200	0.51639700	-1.04782900
H	-0.53152200	0.87661100	2.27939600
H	0.37736900	-1.50185500	1.75478400
H	-3.01573300	-0.76170300	-0.14190900
H	-2.24136900	-1.45992200	-1.55028700
H	-2.28340400	1.08822800	-1.56642500
H	-0.68928500	0.37220300	-1.75434900
H	0.75336900	2.38665400	0.90727200
H	1.75467600	1.54497000	-1.42889700
C	2.14703600	-0.16450300	-0.40742000
O	2.73475500	-1.15500700	-0.29845700

IIc-TS

C	1.16694400	-1.09133200	-0.60515200
C	1.52343900	0.36438500	-0.37919500
C	0.39798300	1.43860900	-0.28153800
H	0.78408600	2.44251600	-0.49111300
C	0.98372700	0.73499700	0.94792600
C	0.24075700	-0.50647900	1.42193900
C	-0.85806000	-0.96302500	0.45496600
H	-1.40563800	-1.82045000	0.87687900
C	-1.81042100	0.19873900	0.11076700
C	-1.03706700	1.22675800	-0.73931400
H	1.58261700	1.30383300	1.66330500
H	-0.12773400	-0.43747200	2.45857700
H	-2.16004600	0.65345300	1.05238700
H	-2.70193300	-0.14814500	-0.43786700
H	-1.56096400	2.19716200	-0.72418700
H	-1.03891800	0.88206900	-1.78921500
H	2.50016200	0.69351600	-0.74462400
H	1.88311800	-1.70027500	-1.17175100
N	-0.15221500	-1.43981400	-0.75035300
H	-0.33305600	-2.40341700	-1.02230600

IIc-P2

C	-1.79328000	-0.09492900	0.54755800
C	-1.17908500	0.09238800	-0.77940000
C	0.13286000	0.84273300	-0.95242800
H	0.17637900	1.44089700	-1.85410000
C	0.10519100	-0.64072200	-1.14600000
C	0.83489700	-1.46484600	-0.14601400
C	1.77449800	-0.93584200	0.63655200
H	2.31593500	-1.56962100	1.33196300
C	2.13019400	0.52985900	0.57977400
C	0.90786500	1.39505900	0.24037200
H	0.13099900	-1.01712300	-2.16268200
H	0.59689000	-2.52247000	-0.08540600
H	2.91050600	0.67734000	-0.17667900
H	2.56096300	0.84975200	1.53123700
H	1.22418600	2.41626000	0.01940300
H	0.26588700	1.45942800	1.12192000
H	-1.90834300	0.21766700	-1.57014200
H	-1.11666800	-0.36102400	1.36652100
N	-3.04740500	0.03018300	0.70297700
H	-3.30374700	-0.14458600	1.67464300

IId-TS

C	-1.39602000	1.09566400	0.12448200
C	-0.18609200	1.65742000	0.07108200
C	1.10574000	0.88742300	-0.13924400
H	1.92648700	1.59524900	-0.25931600
C	0.96947000	0.02323300	-1.37590400
C	0.12834500	-1.00578200	-1.31308800
C	-0.64078800	-1.18301700	-0.02910300
H	-1.17357700	-2.13244000	-0.01718100
C	0.26424600	-1.08181800	1.20856000
C	1.39111500	-0.03638000	1.05808200
H	1.52030600	0.26479500	-2.27900300
H	-0.06018900	-1.66223100	-2.15421600
H	0.69150000	-2.06942200	1.39472700
H	-0.37718900	-0.84738400	2.06130600
H	2.35153700	-0.53291900	0.89577300
H	1.47654600	0.56047700	1.96889400
H	-0.14780600	2.73559000	0.16180100
H	-2.29266900	1.69420100	0.23393900
O	-1.71638100	-0.21829600	0.04550900

C	0.99187200	-1.17203600	-0.71816700
C	1.54677300	0.18170100	-0.35769100
C	0.56074900	1.39287900	-0.22667700
H	1.05372900	2.35452000	-0.41141400
C	1.06922400	0.57336100	0.97488900
C	0.16224100	-0.54918200	1.43714300
C	-0.92747200	-0.88624400	0.46456900
H	-1.56113400	-1.70790500	0.83154400
C	-1.76865200	0.33788600	0.06633800
C	-0.88358900	1.34267600	-0.70151000
H	1.74549100	1.04672900	1.69034100
H	-0.21936900	-0.45886800	2.46432100
H	-2.16439200	0.78776000	0.99131600
H	-2.63309700	0.04567300	-0.55159100
H	-1.31544500	2.35431300	-0.62646500
H	-0.90125300	1.08120200	-1.77414700
H	2.55875100	0.39096000	-0.71523500
H	1.52167500	-1.75066700	-1.48728700
O	-0.32397900	-1.43374600	-0.75559400

IId-P1

C	1.08789700	-0.90963600	-0.47683900
C	1.40674800	0.58955400	-0.55187800
C	0.25222600	1.46587700	-0.14212100
H	0.53737100	2.51082600	-0.21046400
C	1.00751900	0.74291800	0.92759400
C	0.60153500	-0.74304100	0.96135200
C	-0.78268000	-1.10258500	0.36477000
H	-1.20713200	-2.02963900	0.75603200
C	-1.78483900	0.03102200	0.34193000
C	-1.21607500	1.20856500	-0.45328800
H	1.54371600	1.25064900	1.71748000
H	0.97904800	-1.34934800	1.77901100
H	-1.98018500	0.32616200	1.37905000
H	-2.73112800	-0.29977600	-0.09207300
H	-1.78522600	2.11207500	-0.22227600
H	-1.33091000	1.01455700	-1.52126900
H	2.25924600	1.01346000	-1.06619800
H	1.89535200	-1.58063600	-0.76733600
O	-0.20176700	-1.33304700	-0.94763400

IId-P2

C	-1.64627900	0.41007600	0.45971100
C	-1.10852400	-0.69098900	-0.37435400
C	0.13757600	-0.58491400	-1.23635000
H	0.03313300	-1.11974800	-2.17346000
C	0.18104100	-1.43129500	-0.00685000
C	1.03202900	-0.94459700	1.11043800
C	1.60882200	0.25753700	1.11807900
H	2.21857600	0.54847300	1.96737400
C	1.42142400	1.26436400	0.01625700
C	1.05047100	0.62114200	-1.32851500
H	0.12819000	-2.50671700	-0.12215000
H	1.18392100	-1.62124800	1.94475700
H	2.33464500	1.85199500	-0.10664700
H	0.65107600	1.98606900	0.31230200
H	1.97066200	0.27079500	-1.80639700
H	0.61615100	1.37170000	-1.99290600
H	-1.90152200	-1.28965300	-0.80709400
H	-0.96284700	0.86981400	1.19339900
O	-2.79141800	0.77882200	0.38004100
